# Structure and Chemistry of Flat and Stepped Rh Surfaces during NO Dissociation near 1 mbar

**DOI:** 10.1021/jacs.5c18969

**Published:** 2026-04-02

**Authors:** Fernando García-Martínez, Hanna Sjö, Khadiza Ali, Lisa Rämisch, Harald Wallander, Lindsay R. Merte, Zoltan Hegedüs, Johan Zetterberg, Edvin Lundgren, Frederik Schiller, Johan Gustafson, J. Enrique Ortega

**Affiliations:** † 28332 Deutsches Elektronen-Synchrotron DESY, Notkestr. 85, 22607 Hamburg, Germany; ‡ Departamento Física Aplicada, Universidad del País Vasco, 20018 San Sebastián, Spain; § Division of Synchrotron Radiation Research, 209298 Lund University, 22100 Lund, Sweden; ∥ Department of Microtechnology and Nanoscience, 225299 Chalmers University of Technology, Chalmersplatsen 4, 41296 Götenborg, Sweden; ⊥ Department of Physics, 201280 BITS Pilani, Hyderabad Campus, Hyderabad, Telangana 500078, India; # Materials Science and Applied Mathematics, 5264 Malmö University, 20506 Malmö, Sweden; ∇ NanoLund, Lund University, 22100 Lund, Sweden; ○ Division of Combustion Physics, 5193 Lund University, 22100 Lund, Sweden; ◆ Centro de Física de Materiales CSIC/UPV-EHU-Materials Physics Center, Manuel Lardizábal 5, 20018 San Sebastián, Spain

## Abstract

The dissociation of NO is a critical step in its catalytic reduction to N_2_, which is key to automotive exhaust treatment. Here, we examine the role of Rh atomic steps in the NO dissociation reaction under 0.05 mbar NO. We use a Rh crystal sample curved around the (111) direction and ambient-pressure X-ray photoelectron spectroscopy to probe different Rh surfaces subject to the very same reaction conditions. At the dissociation onset, this approach allows us to quantitatively determine the NO species involved in the reaction, and to rationally assess the process in terms of diffusion and dissociation probability at terraces and steps. At a higher temperature we trigger surface oxidation, which begins preferentially on flat Rh(111) and B-type stepped surfaces, as compared to A-type stepped surfaces. Surface X-ray diffraction performed on single crystal samples reveals similar oxide structures at the atomic scale, but while B-type Rh(553) and Rh(111) surfaces do not reconstruct, A-type Rh(223) facet exhibits faceting. These findings underscore the structural sensitivity of NO dissociation and its potential impact on Rh-catalyzed NO reduction.

## Graphical abstract

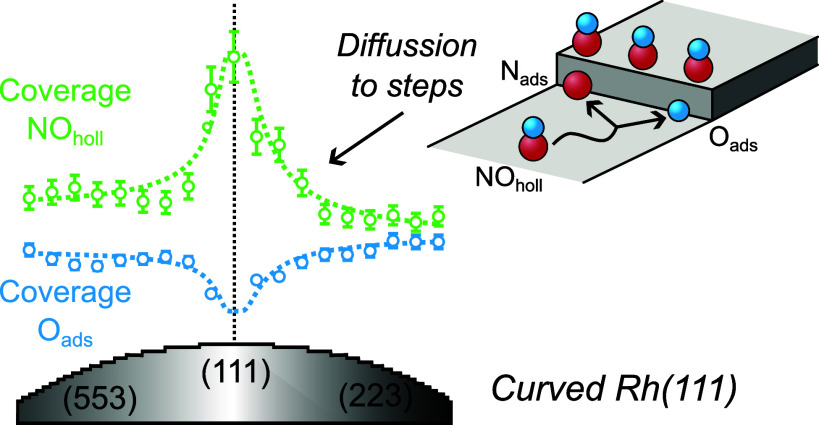

## Introduction

Dissociative adsorption is a key initial step in nearly all relevant gas-surface reactions. A notable example is NO reduction in catalytic converters, where Rh catalysts play a crucial role in reducing exhaust emissions.^1^ Despite its importance, the surface chemistry of NO remains poorly understood, particularly with respect to its structure sensitivity. NO dissociation yields atomic N and O that remain chemisorbed on the surface^2–7^ and requires nearby vacant sites to proceed efficiently.^8^ Consequently, as the surface coverage of NO, N, or O increases, dissociation becomes increasingly inhibited.^9,10^ Because N atoms recombine at much lower temperatures than O atoms,^9,11^ coreactants such as CO or H_2_ are typically used to remove surface oxygen and prevent catalyst poisoning. Higher NO partial pressures further suppress the formation of vacant sites, thereby shifting the onset of NO dissociation and reduction to higher temperatures.^12,13^

Among Pt-group metals, Ir and Rh exhibit the highest activity for NO dissociation,^1,14,15^ with a pronounced sensitivity to the crystal facet. In the case of Rh, the (110) surface is the most active, followed by the (100) facet, while the (111) surface is comparatively less effective.^13,16^ Vicinal surfaces exposing atomic steps are generally more active than flat (111) terraces, as confirmed by ultrahigh vacuum (UHV) experiments and theoretical studies.^4,17–20^ NO adsorbs preferentially at Rh steps rather than at terraces at room temperatures,^19^ although this behavior may differ at higher temperatures.^21^ Moreover, other works report that NO desorption^9^ and NO hydrogenation^19^ are structure insensitive. Overall, this background suggests a complex effect of Rh steps on the NO chemistry, particularly on its dissociation.

While operando experiments are imperative for gaining a comprehensive understanding of the NO chemistry on Rh surfaces, most of the aforementioned NO dissociation studies were performed under UHV conditions or rely on theoretical calculations. Experiments near 1 mbar NO have focused only on Rh(111);^3,12,22,23^ therefore, the role of active Rh steps remains unexplored at this pressure regime. For this purpose, a curved crystal that systematically exposes different facets serves as a convenient sample.^24,25^ In fact, it has been shown that the ability to probe different crystallographic orientations under the same experimental conditions enables a straightforward and accurate assessment of the role of atomic steps in other surface reactions.^26–28^

In this work, we investigate the temperature-dependent evolution of the surface chemistry and the structure of various Rh crystal planes exposed to NO in the mbar regime. First, we employ a curved Rh(111) sample exhibiting surfaces with A-type and B-type steps to monitor the chemical composition of different crystal facets simultaneously with ambient-pressure photoemission. The tunable step-density of the curved surface allows us to quantitatively assess the NO dissociation process right at its temperature onset (100 °C), in terms of diffusion length on terraces and dissociation/desorption probabilities at steps. At higher temperatures (200 °C), complete NO dissociation and desorption lead to the formation of surface oxide patches primarily on (111) terraces and B-steps, while oxygen remains chemisorbed at A-type vicinal surfaces. Lastly, surface X-ray diffraction experiments show a similar surface oxide in all Rh facets, with distinct orientation with respect to the local crystal direction. These observations indicate a strong structure-dependence of NO dissociation, and suggest that NO reduction will also be influenced by the crystal orientation of the surface.

## Results and Discussion

Ambient-Pressure X-ray Photoemission Spectroscopy (AP-XPS) and Surface X-ray Diffraction (SXRD) were employed in this work. On the one hand, AP-XPS was performed using a curved Rh(111) crystal, namely a cylindrical sector centered around Rh(111) [c-Rh(111), see Figure 1b]. The curvature leads to a controlled increase of the vicinal angle α away from the (111) plane located at the crystal center. The design of the c-Rh(111) sample allows to probe either A-steps (square, {100} microfacet, α > 0) or B-steps (triangular, {111} microfacet, α < 0) at each side of the sample.^24,27^ On the other hand, for SXRD experiments we used flat Rh single crystals with (111), (223), and (553) orientations. Rh(111) features large terraces separated by a small density of defects, while the other two surfaces feature relatively narrow 4-atom-wide (111) terraces separated by monoatomic A- or B-steps. The Methods section provides further details about the employed techniques and samples.

**Figure 1:**
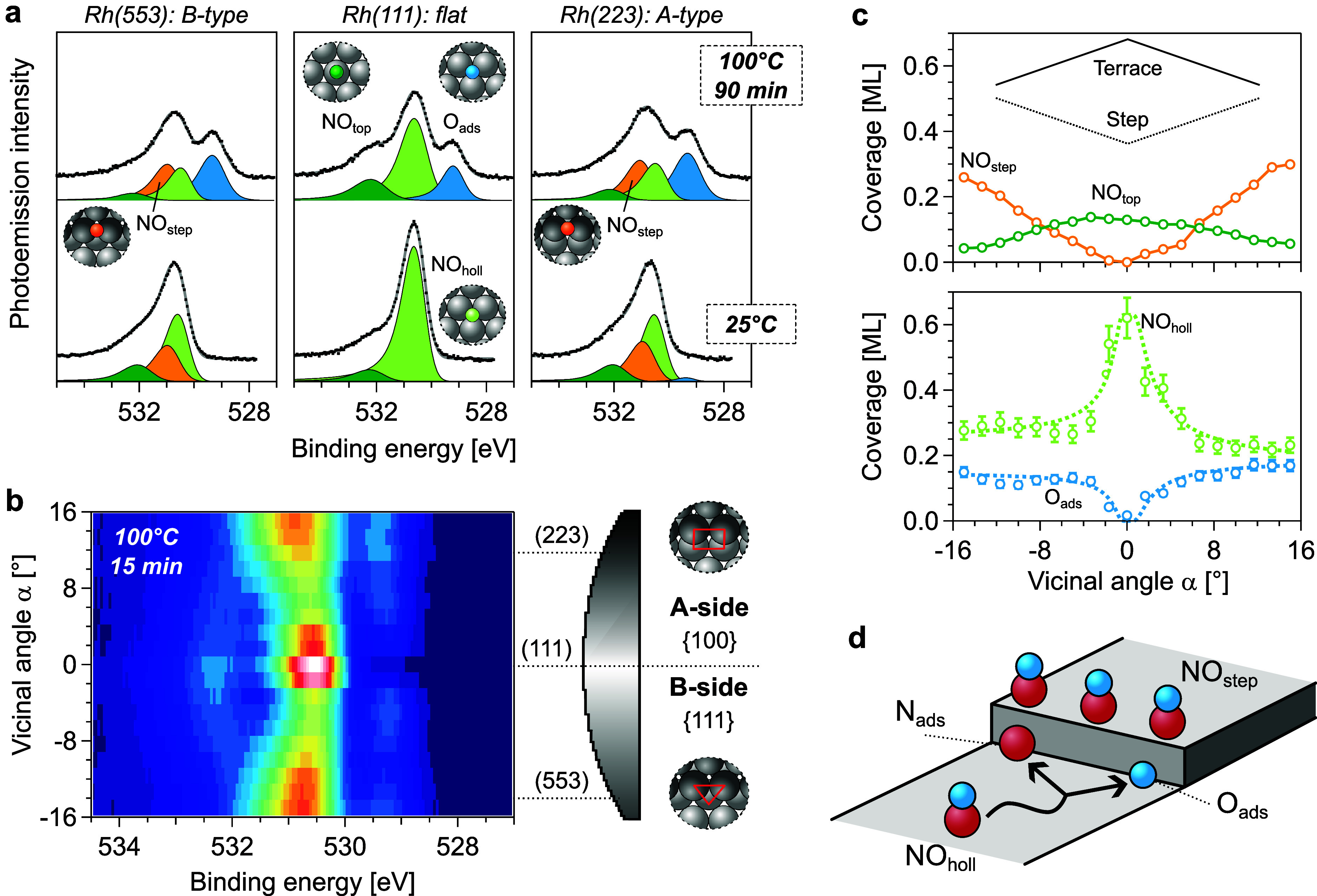
O 1s analysis across the curved Rh(111) crystal at the temperature onset of NO dissociation. (a) O 1s core level measured at the position of the (553), (111), and (223) surfaces of the c-Rh(111) sample. Spectra were acquired at 25 °C (bottom row) and 100 °C (top row, after ≈90 min annealing at 100 °C) under 0.05 mbar NO (0.5 mbar mixture of 10% NO in He). (b) Snapshot of the chemical composition of the curved surface during the early dissociation process. The NO-covered c-Rh(111) sample (sketched on the right side) is annealed at 100 °C for 15 min under the same gas mixture, and then quenched to room temperature to acquire this O 1s α-scan. (c) Coverage variation of individual adsorbates across the O 1s α-scan shown in panel (b). The expected trend of step and terrace species with α is also shown in the top of the panel.^24^ Dashed lines of NO_holl_ and O_ads_ coverages correspond to the diffusion model described in Figure S3. (d) Proposed dissociation mechanism near a step edge, as described in the main text.

### Dissociation Onset at Rh Steps

We first investigate the interaction of NO with the different facets of the c-Rh(111) sample from 25 °C up to the temperature onset of NO dissociation. To this end, the c-Rh(111) sample was exposed to 0.05 mbar NO (0.5 mbar mixture of 10% NO in He) and stepwise heated while monitoring surface species with AP-XPS. Three relevant facets of the curved surface with different atomic sites were probed, namely the flat (111), the A-type (223) and the B-type (553) facets. We focus on the O 1s region in Figure 1, while the corresponding N 1s spectra are shown in Figure S1 of the Supporting Information (SI).

The bottom row of Figure 1a shows the fitted O 1s spectra under 0.05 mbar NO at 25 °C. Molecularly adsorbed NO exhibits two well-resolved peaks in Rh(111), which correspond to NO adsorbed in hollow (NO_holl_, 530.7 eV, main peak) and in top sites (NO_top_, 532.3 eV, smaller peak).^3,12,22^ At the stepped surfaces, a satisfactory fitting cannot be obtained with only two species, and an additional peak in between NO_top_ and NO_holl_ must be added. This new contribution steadily increases with the step density; hence, we ascribe it to molecular NO adsorbed at steps (NO_step_, 531.3 eV), likely at bridge sites.^17,20^ Oxygen species arising from NO dissociation appear at significantly lower binding energies than molecularly adsorbed NO. A single feature arises in the O 1*s* from chemisorbed oxygen, while a doublet is expected for the surface oxide trilayers, therefore these two species are also easily identified.^29–31^

As no peaks arising from NO dissociation products are detected at room temperature, next we annealed the sample in 25 °C steps to trigger the dissociation of NO. When the sample temperature reaches 100 °C (top row of Figure 1a), atomic oxygen anchored at hollow sites (O_ads_) appears at 529.3 eV at the (111) facet^29^ and at 529.4 eV at the stepped surfaces. Therefore, the temperature onset for NO dissociation lies between 75 and 100 °C in 0.05 mbar NO.

Acquiring spectra at three sample positions at 100 °C resulted in a total annealing time of roughly 90 min, during which further reactions may have occurred on other surface regions. To obtain a snapshot of all surfaces under identical reaction conditions, NO dissociation was quenched by annealing a reprepared sample at 100 °C for 15 min under 0.05 mbar NO, followed by rapid cooling to 25 °C without pumping out the gas. The entire curved surface was then probed by acquiring photoemission spectra in small Δα ≈ 2° steps, taking care that no time evolution occurs throughout the acquisition of this α-scan (see Methods section). The O 1s α-scan is shown as a color map in Figure 1b, while corresponding N 1s and Rh 3d data appear in Figure S2 of the SI.

The α-scan allows a direct visualization of binding energy and intensity changes experienced by the different species from the flat (111) center to the stepped edges of the sample. There is a sizable binding energy variation in the NO_top_ terrace species which is likely connected to the α-dependent average strain of terraces in the substrate,^32^ although the possibility of oxygen-induced shifts in the binding energy cannot be excluded. Dissociation of NO at 100 °C did not take place during the exposure of 15 min at the (111) surface; O_ads_ is almost absent around α ≈ 0 in Figure 1b. Nevertheless, 90 min exposure time leads to a considerable amount of O_ads_ (top row of Figure 1a). By contrast, the oxygen content at the stepped surfaces is significant regardless of the annealing time. We therefore conclude that both A- and B-type Rh(111) vicinals are more active than Rh(111) toward NO dissociation under 0.05 mbar NO.

A systematic study of the coverage of the individual adsorbates can be performed after fitting individual spectra of the O 1s α-scan. NO coverages higher than 0.78 ML are unlikely for Rh(111).^33^ Since the Rh(111) spectra shows no signs of dissociation at 25 °C, it is reasonable to assume the same 0.78 ML saturation coverage of NO on Rh(111) under ambient pressure conditions, and use this as NO coverage calibration. The resulting curves are shown in Figure 1c. Note that the step density grows with |α|, in parallel to a decreasing average terrace width. Consequently, the amount of species anchored at steps increases with |α|, while that of species at terraces decreases. NO_step_ and NO_top_ peaks exhibit the characteristic intensity increase/decrease with |α|,^24,34,35^ reflecting the linearly increasing density of steps and decreasing size of terraces across the curved surface. From the slope of the curve, the coverage of approximately one NO_step_ molecule per Rh step atom is obtained, therefore steps are NO-saturated.

Only NO_holl_ exhibits a pronounced change upon annealing (see Figure 1a). Consistently, the NO_holl_ and O_ads_ coverage curves obtained after the 15 min flash at 100 °C (bottom panel of Figure 1c) display a nonlinear and correlated variation as a function of α. This suggests that NO_holl_ is the active species at the onset of NO dissociation, matching well with previous NO reduction experiments on Rh(111) at similar NO pressures.^3^ Since steps remain saturated with NO_step_, the access of NO_holl_ to the step edge is likely blocked from the upper step side. On the other hand, NO_holl_ could reach the step edge from the lower terrace, as sketched in Figure 1d. This interpretation is consistent with calculations of NO dissociation at understep sites on Rh^20,36^ and early experimental observations on Ru.^37^ The direct dissociation of NO_step_ at the Rh steps, followed by the diffusion of NO_holl_ toward step edges to refill empty sites, is an unlikely mechanism.^38^

The correlated α-dependence of NO_holl_ and O_ads_ across the curved surface likely reflects the ability of NO_holl_ molecules to reach the steps. As discussed in Figure S3, the quantitative variation of the O_ads_ coverage with α can be described within a one-dimensional random-walk model for terrace NO_holl_ diffusing toward step sites. The blue line in Figure 1c fits the O_ads_ data using this model, which renders dissociation probabilities σ_dis_ with a reasonable accuracy, namely σ_dis_^A^ = 0.30 ± 0.03 and σ_dis_^B^ = 0.24 ± 0.04 for A- and B-type steps, respectively. Although the O_ads_ and NO_holl_ curves in Figure 1c are qualitatively correlated, the NO_holl_ intensity drop from the (111)-oriented center to the stepped edges is larger. After discarding photoelectron diffraction effects (see Figure S2), we believe that this bigger drop is due to a parallel NO_holl_ desorption process occurring at similar temperatures.^11,39,40^ The light green dashed line in Figure 1c fits the NO_holl_ data with the same random walk model, but considering additional desorption probabilities σ_des_ near A- and B-type steps of σ_des_^A^ = 0.44 ± 0.03 and σ_des_^B^ = 0.37 ± 0.03 (see also Figure S3 of the SI).

The N 1s region was also acquired during the experiments described above (see Figures S1 and S2). However, core-level peaks from the different NO adsorption sites are difficult to resolve due to final state effects.^23^ Moreover, adsorbed nitrogen (N_ads_) is detected mainly at stepped surfaces, yet a clear trend with temperature cannot be observed. Furthermore, N_ads_ is significantly smaller than O_ads_ (see Figure S1), in striking contrast with UHV reports, where N_ads_ and O_ads_ have similar intensity after NO dissociation.^21^ This apparent disagreement is explained by the much higher NO pressure in AP experiments, which shifts the NO dissociation onset to higher temperature.^12,22^ An elevated temperature enhances the N_ads_–N_ads_ recombination toward N_2(g)_, whereas the mbar pressure favors the N_ads_–NO_ads_ reaction toward N_2_O_(g)_,^3^ both explaining the smaller intensity of N_ads_ with respect to O_ads_. These strong dissimilarities between AP and UHV experiments again emphasizes the need of experiments near 1 mbar and above.

### Onset of Surface Oxide Formation

Heating the NO-covered surface beyond 100 °C leads to further NO desorption and dissociation. At 200 °C no more NO is detected on the surface, as judged by a flat N 1*s* core level (see Figure S1). In Figure 2 we show the O 1s region for the (553), (111), and (223) surfaces within the curved crystal at this latter temperature. In the (111) facet (Figure 2a), the contribution from O_ads_ at 529.5 eV has grown considerably relative to spectra at lower temperatures of Figure 1a. Moreover, an additional peak at lower binding energy points to the formation of O–Rh–O surface oxide trilayers (≈528.6 eV). A higher binding energy component is expected close to the energy of O_ads_, which cannot be resolved in this experiment. Since a full trilayer should feature two components of equal height,^31^ we conclude that the surface oxide partially covers the Rh(111) surface and still coexists with O_ads_ under the present conditions.

**Figure 2:**
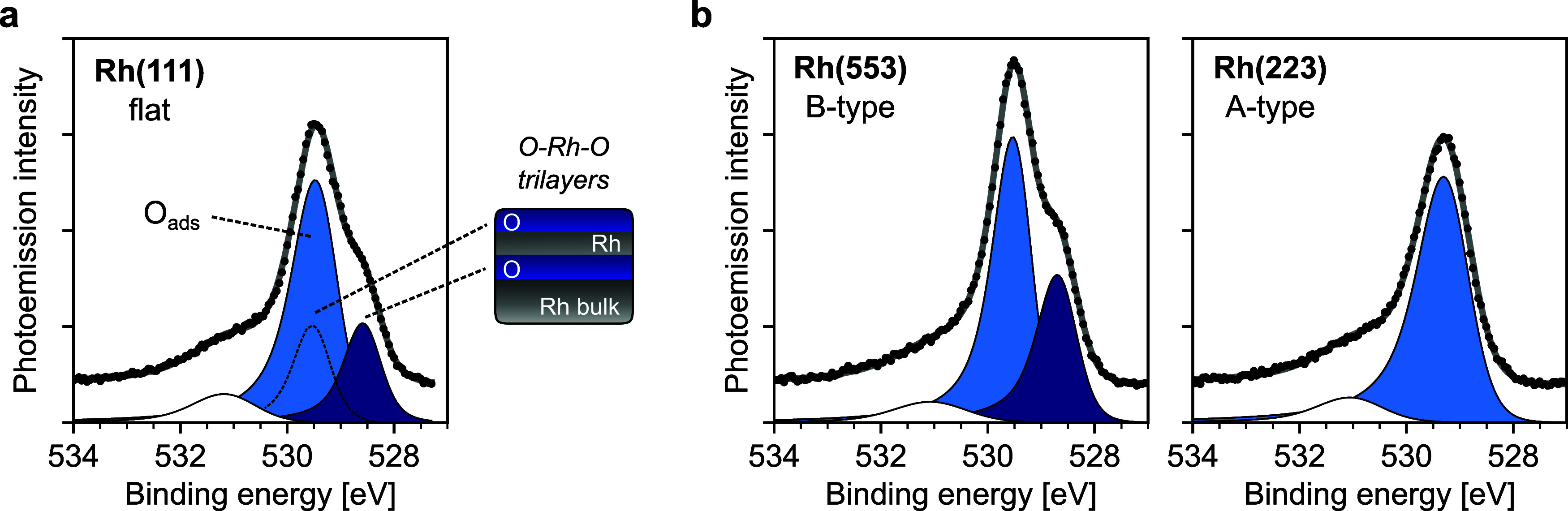
Onset of oxidation at 200 °C on the c-Rh(111) sample. O 1s photoemission core level at 200 °C measured at the (a) (111) and (b) (553) and (223) facets of the c-Rh(111) sample, together with a sketch of the surface oxide trilayers next to the (111) spectra. Spectra were acquired under 0.05 mbar NO (0.5 mbar mixture of 10% NO in He). The surface oxide observed at this temperature is amorphous, as judged by the SXRD data shown in the next section. The peak near 531 eV is discussed in Figure S4 of the SI, together with an α-scan obtained under the same conditions as those of this Figure.

The O 1s core level at 200 °C for the (553) and (223) facets of the c-Rh(111) is shown in Figure 2b, featuring strong A/B asymmetries. On the one hand, the characteristic doublet of the Rh surface oxide is observed for Rh(553), where the large O_ads_ contribution also suggests an incomplete oxide trilayer. On the other hand, at the (223) surface only O_ads_ is detected. The α-scan shown in Figure S4 shows mainly O_ads_ on the A-side of the crystal, while the two components of the surface oxide are easily distinguished near the (111) surface and across the B-side of the c-Rh(111) crystal. This confirms that the surface oxide forms more easily in B-steps than A-steps, agreeing well with previous oxidation experiments using O_2_.^41^ In our previous CO oxidation experiments at ≈1 mbar using the same c-Rh(111) sample, we concluded that the lack of surface oxide at B-steps is a result of their larger activity toward CO oxidation as compared to A-steps, where almost a full trilayer was observed.^27^ Therefore, the higher tendency of B-steps toward surface oxidation by NO agrees well with B-steps being more active than A-steps.

### Structural Analysis of the Surface Oxide

To complement the AP-XPS measurements and study the structure of the surface oxide, we carried out separate SXRD measurements on flat Rh(111), Rh(553), and Rh(223) single crystals. Clean surfaces were exposed to 0.05 mbar NO at room temperature, and SXRD images were acquired in 50 °C steps to monitor the emergence of the surface oxide and its further evolution. Rotational scans around the axis perpendicular to the surface are performed to map the reciprocal space. The real and reciprocal spaces of the different surfaces are described in Figure S5, whereas observed Bragg spots are shown in Figure S6. We focus on the *k*-direction in the 0*kl* plane. For each surface we define the scattering vectors *Q*_*k*_ and *Q*_*l*_ along *k* and *l*, respectively, and *Q*_*x*_ perpendicular to *Q*_*k*_.

SXRD images belonging to the *k*-direction of Rh(111) are shown in Figure 3a. The reciprocal *hk* plane and *k*-direction of Rh(111) are respectively sketched in panels [Fig fig3]b,c. Diffraction rods arise perpendicular to the surface due to the truncation of the 3D crystal symmetry at the catalyst surface. Crystal truncation rods (CTRs) intersecting Bragg spots or superstructure rods not intersecting them are therefore expected along *l*, as sketched in Figure 3c.

**Figure 3:**
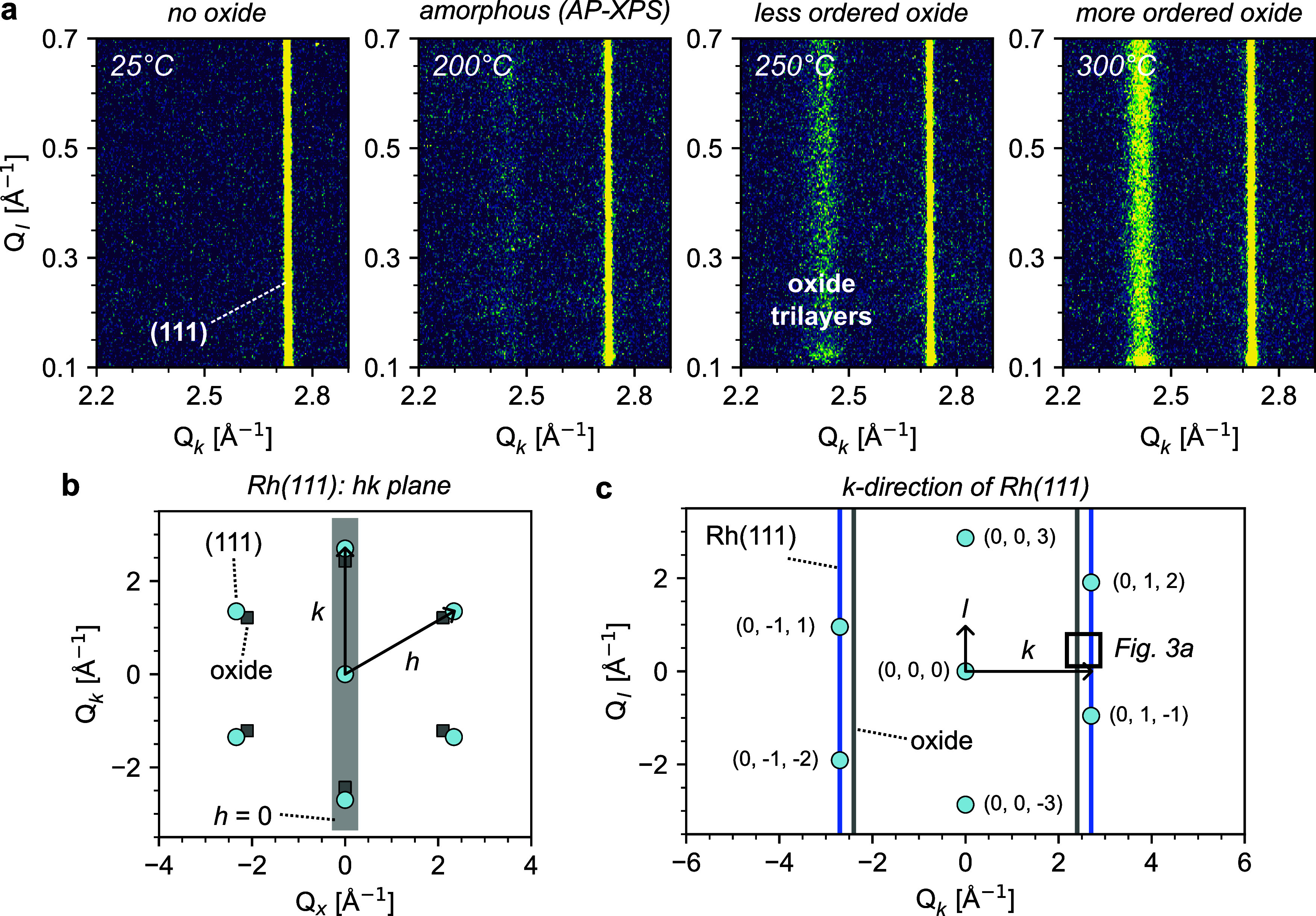
Surface structure of Rh(111) after surface oxidation with NO. (a) SXRD images obtained along the (0, 1, *l*) direction extending from the (0, 1, 2) Bragg reflection of a Rh(111) single crystal. Diffraction patterns were acquired under 0.05 mbar NO at 68 keV, before (25 and 200 °C) and after (250 and 300 °C) ordered surface oxide formation. Rods are labeled according to their respective surface or structure. In this case, all rods are perpendicular to the surface, with no measurable tilt. (b) *hk* plane of Rh(111), showing the lattice points of Rh(111) as well as its surface oxide. We also illustrate the scanned slice corresponding to the *k*-direction at *h* = 0. (c) Further details on the 0*kl* plane of Rh(111), namely the *k*-direction. Diffraction rods arising along *l* for Rh(111) and its surface oxide are also illustrated. See Figure S7 in the SI for entire SXRD images.

At room temperature, the data shows a single rod at *Q*_*k*_ ≈ 2.7 Å^–1^. As sketched in Figure 3c, this matches well with the expected position for a CTR of Rh(111).^31^ Further heating of the sample to 250 °C causes the appearance of an additional rod at about *Q*_*k*_ ≈ 2.4 Å^–1^, marking the onset of ordered surface oxide formation. This rod is consistent with the 9 × 9 surface oxide of Rh(111) formed upon oxidation with O_2_,^31^ hence the surface oxide formed upon NO exposure is quite similar. Heating to 300 °C causes the oxide rod to grow in intensity, showing that O–Rh–O trilayers become more ordered as the sample temperature increases. No strong signatures of surface oxide are observed below 250 °C with SXRD; hence, the surface oxide observed in AP-XPS at lower temperatures is likely amorphous.

Corresponding SXRD data for Rh(553) and Rh(223) single crystals are shown in Figures 4 and 5. Surfaces were exposed to 0.05 mbar NO at 25 °C and then heated in steps of 50 °C up to a maximum temperature of 350 °C. For simplicity, we only show the measurements at 25 °C just after introducing the NO, at 350 °C, and at 25 °C again after the annealing. The surface structure and *k*-direction for both facets are respectively sketched in panels a and b of Figures 4 and 5, respectively. (111) coordinates are used to simplify the comparison between different facets. In the vicinal surfaces, **a**_**1**_ is defined along the step edge, while **a**_**2**_ accounts for the terrace width. In reciprocal space, *k* (**b**_**2**_) is perpendicular to the step edge, and *l* (**b**_**3**_) is perpendicular to the surface. The scattering vector has no contributions from *h* (**b**_**1**_) along the *k*-direction.

**Figure 4:**
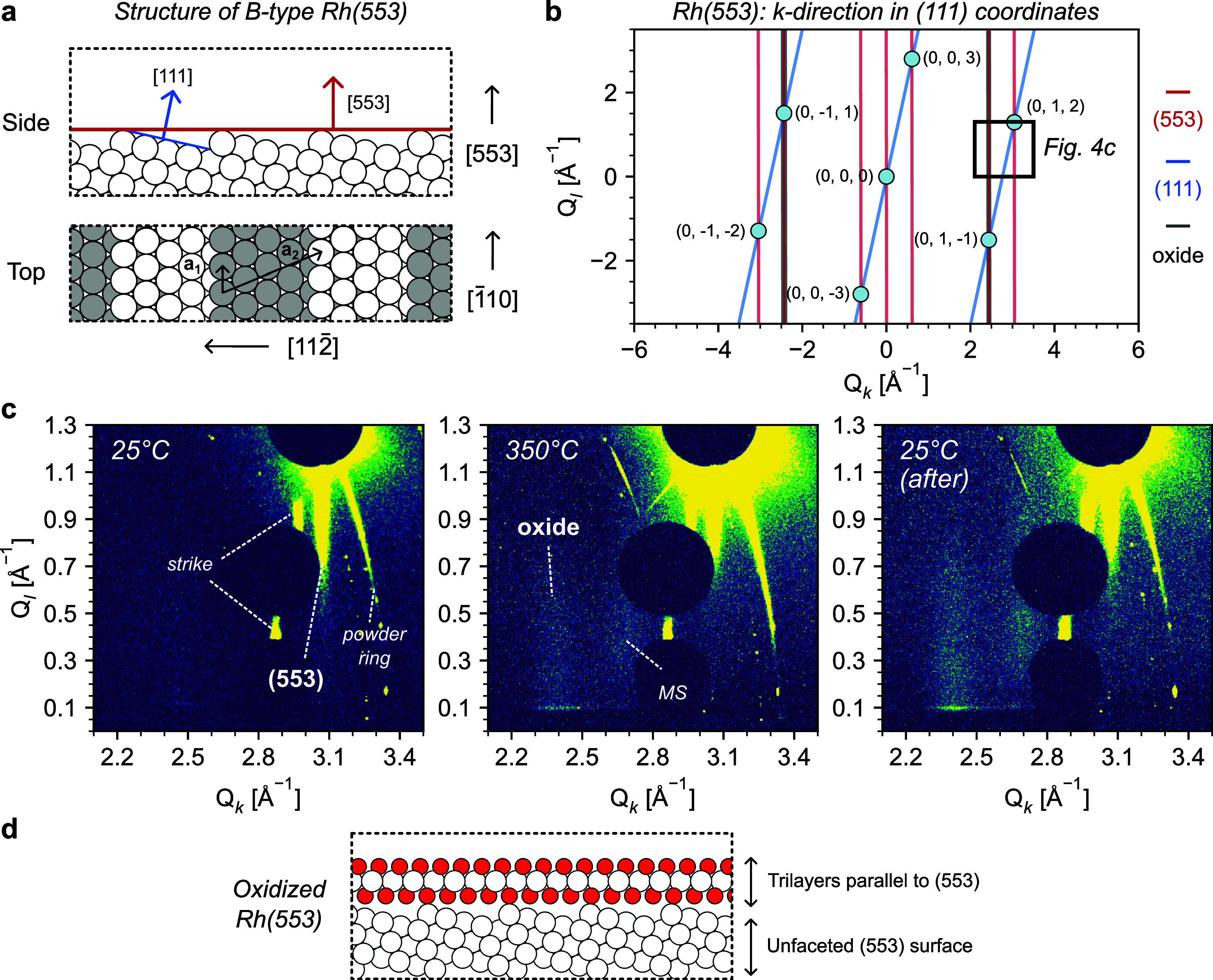
Surface structure of Rh(553) after surface oxidation with NO. (a) Top and side view of a Rh(553) surface, showing in-plane vectors **a**_**1**_ and **a**_**2**_ and out-of-plane [111] and [553] vectors. (b) *k*-direction of Rh(553) showing observed Bragg spots using (111) coordinates. Vertical rods arising from Rh(533) and tilted rods arising from Rh(111) are also sketched. (c) SXRD images obtained using a Rh(553) single crystal along the *k*-direction. Perpendicular rods arise from the (0, 1, 2)_111_ Bragg spot. Rods are labeled according to their corresponding surface or structure. No measurable tilt is observed. (d) Sketch of the surface oxide of Rh(553) upon oxidation with NO. SXRD images were acquired at 68 keV after oxidizing the crystal under 0.05 mbar NO at 25 and 350 °C, and again at 25 °C after annealing the sample. See Figures S8–S9 in the SI for full SXRD images, and the text for more details regarding the multiple scattering (MS) and strike rod of Rh(553).

**Figure 5:**
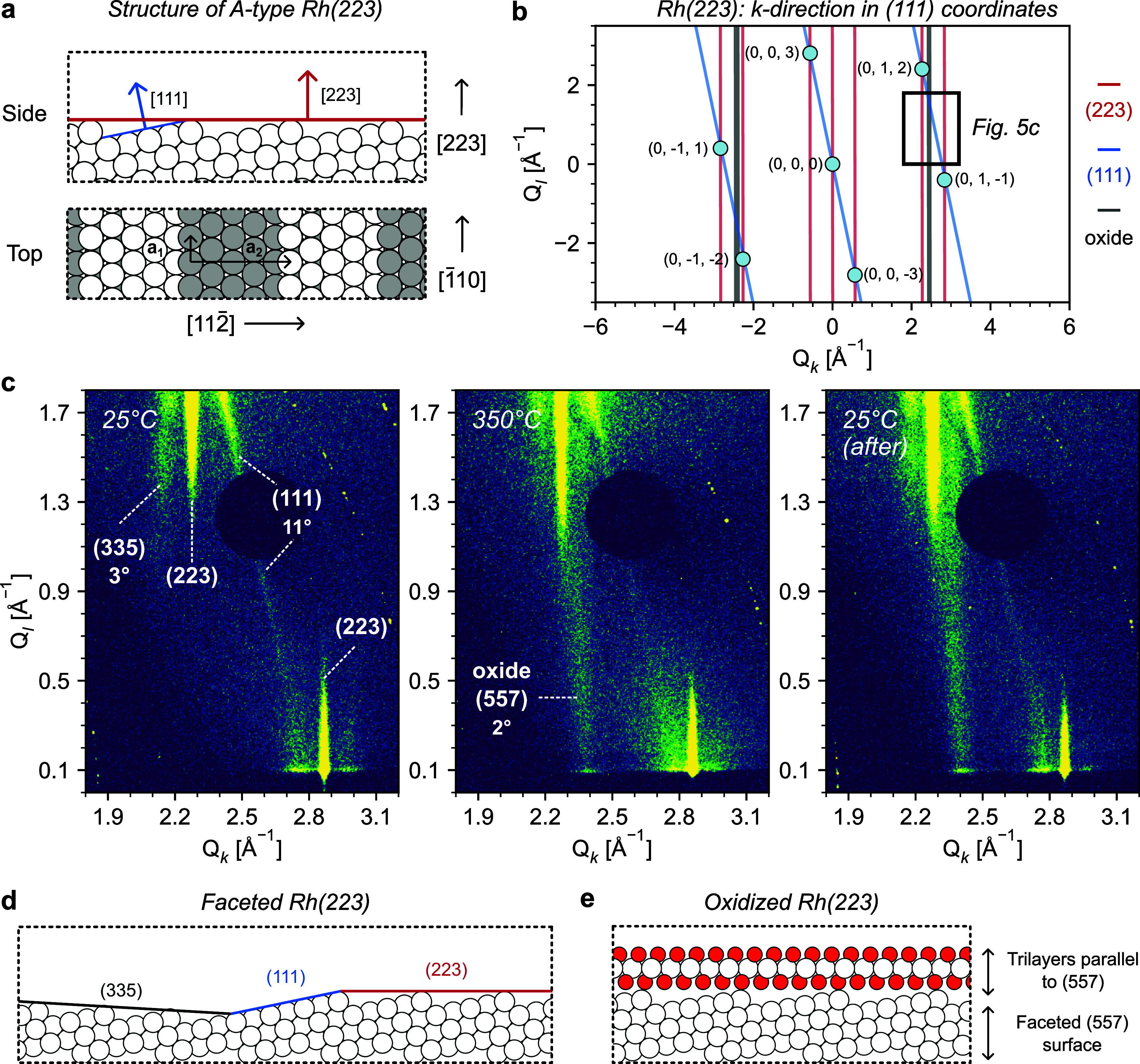
Surface structure of Rh(223) after surface oxidation with NO. (a) Top and side view of a Rh(223) surface, showing in-plane vectors **a**_**1**_ and **a**_**2**_ and out-of-plane [111] and [223] vectors. (b) *k*-direction of Rh(223) showing observed Bragg spots using (111) coordinates. Vertical rods arising from Rh(223) and tilted rods arising from Rh(111) are also sketched. (c) SXRD images obtained using a Rh(223) single crystal along the *k*-direction. Perpendicular rods arise from the (0, 1, 2)_111_ Bragg spot. We also observe reflections extending from the (0, 1, −1)_111_ spot, likely arising from the edges of the sample. Rods are labeled according to their corresponding surface or structure, with tilt indicated if not perpendicular. (d) Structure of Rh(223) after cleaning and (e) upon oxidation with NO. SXRD images were acquired at 68 keV after oxidizing the crystal under 0.05 mbar NO at 25 and 350 °C, and again at 25 °C after annealing the sample. See Figures S10–S11 in the SI for full SXRD images.

Diffraction rods are always perpendicular to the diffracting surface, therefore all rods will be vertical for perfect (553) or (223) surfaces. In the case of faceted surfaces, additional rods will appear perpendicular to the new facets. For vicinal (111) surfaces, faceting typically results in extended (111) terraces. Additional rods are hence expected in the (111) direction, as sketched in Figures 4b and 5b. The tilt of these (111)-oriented rods corresponds to the vicinal angle, namely 12.3° and 11.4° for the (553) and (223) surfaces, respectively.

Starting with the Rh(553) images of Figure 4c, the data at 25 °C shows a strong vertical CTR extending from the (0, 1, 2)_111_ Bragg reflection, indicating a nonfaceted Rh(553) surface. The Bragg reflection as such is blocked by a circular beamstop to protect the detector. Similarly, other parts of the detector, where Bragg reflections appear during a scan, are blocked. Unfortunately, for vicinal surfaces, these appear close to each other and limit the view. Extending from the Bragg reflection, we also find parts of a powder ring originating from defects, typically, at the edge of the sample. One additional strike, better seen in Figure S8, is also observed. We believe this arises from multiple scattering (MS) effects, and not from surface faceting. MS is typically not seen in SXRD, and further discussion on this complicated phenomenon is beyond the scope of this paper and will be the topic of a future publication.

When heating to 350 °C, a superstructure rod appears at 2.4 Å^–1^, revealing the formation of the surface oxide. There is another superstructure rod at 2.7 Å^–1^, which we also believe to correspond to the oxide, but appearing due to MS. The oxide rods are vertical, suggesting a carpet-like growth on the nonfaceted (553) surface, as sketched in Figure 4d. Moreover, these oxide rods are less sharp than for the (111) surface. Corresponding oxide rods in directions more along the step edges are sharper than oxide rods along the *k*-direction for Rh(553), although still less sharp than for the (111) surface (see Figure S9 of the SI). Previous DFT calculations have shown that the trilayer Rh oxide is rather stable on its own,^31^ and the interaction with the Rh substrate is expected to be limited. Hence, on stepped surfaces, we expect the oxide film to stretch between the steps with relatively small, but still significant, relaxations following the terraces. These relaxations will induce a slight variation in the atomic distances, specifically in the direction perpendicular to the step edges, in agreement with the limited sharpness of the oxide rods, especially in this direction.

In Figure 5c we show the corresponding data from Rh(223), focusing on the space between the (0, 1, 2)_111_ and (0, 1, −1)_111_ Bragg reflections. At 25 °C, before heating, we observe vertical rods at *Q*_*k*_ ≈ 2.3 and ≈2.9 Å^–1^, corresponding to the Rh(223) surface. Moreover, there is one rod tilted by about 11°, connecting both Bragg reflections as in Figure 5b. This shows that a portion of the Rh(223) surface has undergone faceting toward extended (111) terraces. To retain the macroscopic surface orientation, these larger (111) facets need to be compensated by areas of smaller terraces. Hence, there is another additional rod, leaning in the other direction by ≈3°, in agreement with the presence of (335) facets. Such faceting is shown in Figure 5d. As shown in Figure S10, these facets were present already after cleaning. This may have been expected according to a previous study, showing that a perfectly ordered Rh(223) surface is difficult to obtain even in the 10^–10^ mbar regime.^42^

After heating Rh(223) to 350 °C the appearance of a superstructure at *Q*_*k*_ ≈ 2.4 Å^–1^ again reveals the formation of a surface oxide. Similarly to Rh(553), the diffraction pattern intensifies after cooling to room temperature. Also in line with the (553) results, oxide rods of Rh(223) along the *k*-direction are less sharp than for Rh(111) and than for the Rh(223) along directions more parallel to the step edges (see Figures S9 and S11 of the SI). As mentioned above, this agrees well with carpet-like oxide growth.

There is, however, a strong difference between the oxides on Rh(553) and Rh(223). For the B-type (553) surface, the oxide grows parallel to the (553) surface itself, while the oxide rod of the A-type Rh(223) facet is leaning about 2° in the direction toward the (111) rod. This suggests that the oxide grows on (557) facets, rather than the unreconstructed (223) surface, as depicted in Figure 5e. To understand this difference, we compare the lattice dimensions of the surface oxide with the step-to-step distance. We define for this the interatomic row distance for the Rh(111) surface oxide (*d*_SurfOx_ = 3.0√3/2 = 2.6 Å).^31^ On one hand, the step-to-step distance of Rh(553) is 10.4 Å, matching closely the distance of four oxide rods (4*d*_SurfOx_ = 10.4 Å). On the other hand, the step-to-step distance is 11.1 Å for Rh(223), which matches with 4 or 5*d*_SurfOx_ significantly less well. For Rh(557), with one more atomic row per terrace, the match is slightly better with 13.5 Å vs 5*d*_SurfOx_ (13.0 Å), providing a driving force for a reconstruction of the substrate to (557) facets. Driving the faceting one step further, the match is even better between the (334) facets of 18.2 Å and 7*d*_SurfOx_ (18.3 Å). Each faceting step, however, also results in a larger surface area, and hence an increased total energy. Our findings suggest that the stabilization of Rh(557) facets arises from an equilibrium between the energetic gain due to improved substrate-oxide lattice matching and the energy cost associated with increased surface area.

In a previous study of the oxidation of Rh(553) by O_2_,^43^ significant step bunching occurred in order to provide extended (111) terraces for the surface oxide to grow on. In this work, no such faceting would occur when oxidizing with 0.05 mbar NO, whereas on Rh(223) oxidation seems to occur forming (557) facets. This difference may be explained by behavior at lower coverages. Prior to the formation of the surface oxide O_2_ induces a reconstruction into (331) and (111) facets, where the step edges are covered by a 1D oxide.^43^ The match between the (331) facets and the surface oxide is very poor, so the growth of the surface oxide will most likely start on the (111) facets. Once these are filled, it is difficult to return to a (553), but to maximize the oxygen coverage, the step bunching is completed resulting in only (111) equivalent facets covered by the surface oxide. With NO, on the other hand, we do not see any faceting at low coverages. Hence, when the oxide growth starts, it fits nicely to the existing nonreconstructed (553) surface. For Rh(223) the story is similar. There is no significant faceting at low coverages, and the oxide growth can easily induce a reshaping of the facets to include one more atomic row.

Another notable difference between this study with NO and the oxidation with O_2_ in ref 43, is that in the present study, we exposed the surface to NO at room temperature and stepwise increased the temperature to 350 °C, while the oxidation in O_2_ occurred directly at 550 °C. It may very well be that the lower temperature, especially in the beginning of the oxidation process, limits the faceting.

## Conclusions

We have studied NO dissociation at Rh(111) and its A/B vicinals at 0.05 mbar NO using AP-XPS and a curved Rh(111) crystal. Our systematic step-density-dependent study at the temperature onset of NO dissociation (100 °C) supports that NO adsorbed at hollow terrace sites diffuses toward the understep region and dissociates there, while the upper step edge remains fully NO-saturated. The AP-XPS analysis of the entire curved surface at the early dissociation stage allows a quantitative determination of the dissociation and desorption probabilities at A- and B-type steps. Further heating to 200 °C under 0.05 mbar NO leads to complete NO dissociation/desorption and the formation of a surface oxide at (111) terraces and B-type surfaces. No surface oxide was observed on A-type surfaces at this temperature by AP-XPS, hence the onset temperature for surface oxidation is higher for Rh A-type steps than for B-type steps and (111) terraces.

We also studied the structure of flat Rh(111), B-type Rh(553), and A-type Rh(223) surfaces upon NO dissociation using SXRD at 0.05 mbar NO. A trilayer surface oxide, similar to that previously observed during oxidation with O_2_, is detected on all three surfaces. On Rh(111), the agreement with previous studies with O_2_ is excellent. For Rh(553), we find that oxidation with NO results in carpet-like oxide growth, with the substrate retaining the (553) orientation. By contrast, oxidation of Rh(553) with O_2_ occurs in conjunction with step bunching, creating large (111) terraces covered by surface oxide. For Rh(223), oxide growth is also carpet-like, but the substrate undergoes slight faceting into the (557) orientation, with terraces one row wider than those of the original (223) surface. We attribute this faceting to a better match between the oxide lattice and the step-to-step distance.

Our results demonstrate a strong structural dependence of NO dissociation on Rh model catalysts, which is expected to directly affect the activity of the same surfaces under NO reduction conditions. Future experiments are planned to further investigate the structural dependence of NO surface chemistry on Rh and Ir.

## Methods

### Curved Rh(111) Crystal and the α-Scan Approach

The c-Rh(111) sample is thoroughly described in refs 24,27 . As sketched in Figure 1b, it features the (111) plane at the apex of the crystal. Its cylinder axis is parallel to the [11̅0] direction, leading to surfaces with a smooth increase of either A-type ({100} microfacet) or B-type ({111} microfacet) close-packed steps as one departs from the center of the crystal. The vicinal angle α is proportional to the step density. Since α corresponds to the angle between a vicinal surface and the high-symmetry (111) facet, α is often a more visual parameter to describe a vicinal surface than the step density. Vicinal angles up to α = ±15° can be probed using this sample, allowing to reach the A- and B-stepped (223) and (553) planes, with α = +11.4° and −12.3°, respectively. One could technically probe the A-type (335) at α = 14.4°, although this is very close to the crystal edge. Instead, we focused on the (223) surface, which has the same terrace width as the (553) plane (4-atom-wide terraces). The curved sample was prepared by several cycles of Ar^+^ sputtering, O_2_ annealing and high-temperature flashes. This yielded a contaminant-free surface with the expected variation of the step density across the sample.

### Ambient-Pressure X-ray Photoemission, AP-XPS

AP-XPS measurements were carried out at the HIPPIE beamline (Max IV synchrotron, Lund, Sweden).^44^ Experiments were acquired with a photon energy of 680 eV at normal emission and 55° incidence angle. Spectra were normalized at the lower binding energy side of the spectra. Binding energy scale was referenced to the Fermi level. We estimate a total resolution of 70 meV at 680 eV and 25 °C. The beam size was estimated to be 25 × 60 μm^2^ (*V* × *H*). The fingerprint in the vertical direction of the small beam spanned over an interval of <0.1°, allowing to probe well-defined crystallographic orientations across the curved sample. Since steps were oriented parallel to the beam, this yielded a horizontal projection of ≈90 μm along the direction of the steps. An example of the α-scan approach with AP-XPS is presented in Supporting Video 1.

Experiments were carried out in 0.05 mbar NO (0.5 mbar mixture of 10% NO in He, flow of 1.5 mL/min). No NO_(g)_ features were observed in XPS due to the low partial pressure of NO. As shown in Figure S12, we observed no sizable change of the spectra upon beam irradiation, hence we discard having significant beam damage effects (i.e., beam-induced NO dissociation) during the time scale of the AP-XPS measurements. Coverages were calibrated assuming that the O 1*s* spectra of the (111) plane correspond to 0.78 ML under 0.05 mbar NO at 25 °C.^33^ The coverage analysis roughly matched a full coverage of the steps, corresponding to a NO_step_ molecule per Rh step atom. As discussed in Figure S2, spectra at different kinetic energies suggest that there is no appreciable photoelectron diffraction effects across the curved sample.

The α-scans shown in Figures 1b and S2 require roughly 8 h of acquisition time, therefore one must verify that no time evolution occurs during their acquisition. To this end, spectra at selected facets were obtained at the beginning, middle and end of the α-scans. No significant change was observed, confirming that rapid cooling effectively suppressed further surface reactions and that the variations observed in the α-scan arise solely from differences in crystal orientation.

Peak fitting was performed using dedicated Python scripts based on Python’s *lmfit* package.^45^ Peaks in the N 1s region showed a significant metallic character, hence markedly asymmetric Doniach-Sunjic lineshapes were utilized.^46^ On the other hand, the asymmetry of the O 1s contributions was not so strong, hence a simpler asymmetric pseudo-Voigt profile was chosen. Further data and image processing was carried out with Igor Pro (WaveMetrics, Inc.) and Inkscape (Inkscape Project) software.

### Surface X-ray Diffraction, SXRD

SXRD measurements were carried out at beamline P21.2 of Petra III synchrotron at DESY (Hamburg, Germany).^47^ The beam energy was 68 keV, and its size was estimated to be 15 × 4 μm^2^ (*H* × *V*). Extreme grazing incidence geometry (0.05°, below the critical angle of Rh) was employed to observe the surface-sensitive CTRs. Rh single crystals were cleaned similarly to as described above, and surface orientation was checked with SXRD under vacuum before gas dosing. Data and image processing was carried out with Python and Inkscape (Inkscape Project) software.

We used a specially designed chamber for the SXRD experiments. The base pressure of the chamber was in the 10^–6^ mbar range. The chamber was backfilled with the gas mixture with virtually no flow. The gas mixture was leaked toward a Mass Spectrometer (MS) operating in vacuum to probe its composition. Sample was heated using a bora-electric heater, and sample temperatures were estimated using a surface temperature versus heater current curve obtained from a different sample. A cylindrical Be tube was used as part of the chamber for good X-ray transmission. The photon detector was placed in air approximately 1.7 m after the chamber, as calibrated using a LaB_6_ sample using PyFAI.^48^ A Varex Imaging XRD 4343CT detector with 2880 × 2880 pixels with 150 × 150 μm^2^ pixel size was employed. Rocking scans were performed rotating approximately 110° around the surface normal. The median image of each rocking scan was subtracted from individual images as background. For each set of images (e.g., those of Figures 3a or 4c), a common intensity range was chosen to enhance the observed rods.

Each Rh surface was defined by two in-plane vectors (**a**_**1**_ and **a**_**2**_) and an out-of-plane vector (**a**_**3**_), from which the reciprocal vectors **b**_**i**_ are calculated. For a (111) fcc surface, the real vectors **a**_**1**_ and **a**_**2**_ are symmetrically equivalent. Therefore, diffraction arising at directions defined by the reciprocal vectors **b**_**1**_ and **b**_**2**_ usually provide the same information in Rh(111). However, **a**_**1**_ and **a**_**2**_ are not symmetrically equivalent in Rh(553) and Rh(223) (see Figure S5), thereby diffraction arising in the direction of **b**_**1**_ and **b**_**2**_ provides different information.

We define **a**_**1**_ along the [1̅10] direction for the three Rh facets, corresponding to the step edges in stepped surfaces. Diffraction along **b**_**1**_ is hence useful to monitor structures along step edges. Conversely, **a**_**2**_ is defined intersecting the step edge to account for the terrace width of the stepped surface. Diffraction along **b**_**2**_ is hence informative about faceting of step bunching that are closely related to the terrace width of a vicinal surface.

The real lattice vectors **a**_**i**_ and the module of the corresponding reciprocal vectors **b**_**i**_ used for Rh(111), Rh(553) and Rh(223) are summarized below. A lattice constant of *a*_0_ = 3.803 Å for Rh was employed.Lattice vectors [Å]Rh­(111)Rh­(223)Rh­(553)
**a_1_** ·(*a*_0_/2)(1̅,1,0)(1̅,1,0)(1̅,1,0)
**a_2_** ·(*a*_0_/2)(1,0,1̅)(3,3,4̅)(3,0,5̅)
**a_3_** ·(*a*_0_/2)(1,1,1)(2,2,3)(5,5,3)
Vector module [Å^–1^]Rh­(111)Rh­(223)Rh­(553)|b_1_|2.702.342.51|b_2_|2.700.570.61|b_3_|0.950.400.22


Detector pixel images were transformed into scattering vector units (*Q*-coordinates, Å^–1^) following the procedure described in ref 49. The vertical axis of the transformed detector image corresponds to the out-of-plane scattering vector *Q*_*l*_, which is related to *l* (**b**_**3**_), while the horizontal axis corresponds to the in-plane scattering vector *Q*_*r*_. The latter depends on both *h* and *k* (**b**_**1**_ and **b**_**2**_), and can be therefore decomposed into *Q*_*x*_ and *Q*_*k*_ components. During a rocking scan around the surface normal, each Bragg spot appears at a specific angle and is characterized by distinct *Q*_*r*_ and *Q*_*l*_ values. This spans a 3D matrix—which corresponds to the reciprocal lattice randomly rotated around the surface normal. To have a common reference coordinate system, this matrix was rotated so that the *Q*_*k*_ component of *Q*_*r*_ matches the direction defined by **b**_**2**_. Finally, the values of *Q*_*i*_ are normalized by **b**_**i**_ to convert the *Q*-coordinates into Reciprocal Lattice Unit (RLU) coordinates. This facilitates the identification of the scattering vector associated with each Bragg reflection.

We observed a different temperature onset for oxide formation in SXRD and AP-XPS. This most likely arises because diffraction techniques require long-order structures, while any species in the surface, either amorphous or ordered, can contribute to the photoemission signal. Since we focused on qualitatively describing the process of NO dissociation, this offset is not relevant for the present study.

## Supplementary Material





## Notes

Illustration of the α-scan approach combining curved crystals and AP-XPS (Videos S1) (MP4)
O 1s and N 1s core level regions measured at the (111), (223), and (553) facets of the curved Rh(111) sample under 0.05 mbar NO at 25, 100, and 200 °C (Figure S1); corresponding N 1s and Rh 3d α-scans obtained at the temperature onset of the NO dissociation (100 °C for 0.05 mbar NO) (Figure S2); diffusion model of NO_holl_ toward the under-step reaction resulting into dissociation to O_ads_ (Figure S3); O 1s α-scan across the curved Rh(111) sample after surface oxidation at 200 °C under 0.05 mbar NO (Figure S4); real and reciprocal surfaces of Rh(111), Rh(223) and Rh(553) (Figure S5); observed Bragg spots of Rh(111), Rh(223), and Rh(553) (Figure S6); additional SXRD images obtained for Rh(111), Rh(553), and Rh(223) (Figures S7–11), and assessment of beam damage during AP-XPS experiments (Figure S12) (PDF)
