# Supplementary material for: Structure and Chemistry of Flat and Stepped Rh Surfaces during NO Dissociation near 1 mbar
Source: J Am Chem Soc. 2026 Apr 2;148(14):14824–34. doi: 10.1021/jacs.5c18969 (PMC13088247; doi:10.1021/jacs.5c18969)
Supplement: Supplementary file 2 [file ja5c18969_si_002.pdf]

Supplementary Information: Structure and chemistry of flat and stepped Rh surfaces during NO dissociation near 1 mbar

Fernando García-Martínez[1,2*], Hanna Sjö[3], Khadiza Ali[4,5], Lisa Rämisch[3], Harald Wallander[6,7], Lindsay R. Merte[6,1], Zoltan Hegedüs[1], Johan Zetterberg[8], Edvin Lundgren[3,7], Frederik Schiller[9], Johan Gustafson[3*] and J. Enrique Ortega[2,9*]

[1]Deutsches Elektronen-Synchrotron DESY, Notkestr. 85, 22607 Hamburg, Germany.
[2]Departamento Física Aplicada, Universidad del País Vasco, 20018, San Sebastián, Spain.
[3]Division of Synchrotron Radiation Research, Lund University, 22100, Lund, Sweden.
[4]Department of Microtechnology and Nanoscience, Chalmers University of Technology, Chalmersplatsen 4, Götenborg, 41296, Sweden.
[5]Department of Physics, BITS Pilani, Hyderabad Campus, Telangana, 500078, India.
[6]Materials Science and Applied Mathematics, Malmö University, 20506, Malmö, Sweden.
[7]NanoLund, Lund University, 22100, Lund, Sweden.
[8]Division of Combustion Physics, Lund University, 22100, Lund, Sweden.
[9]Centro de Física de Materiales CSIC/UPV-EHU-Materials Physics Center, Manuel Lardizábal 5, San Sebastián, 20018, Spain.

*Corresponding author(s). E-mail(s): fernando.garcia-martinez@desy.de; johan.gustafson@fysik.lu.se; enrique.ortega@ehu.eus;

Contributing authors: hanna.sjo@fysik.lu.se;
khadiza.ali@gmail.com; lisa.raemisch@gmail.com;
gna10hwa@student.lu.se; lindsay.merte@mau.se;
zoltan.hegedues@desy.de; johan.zetterberg@forbrf.lth.se;
edvin.lundgren@fysik.lu.se; frederikmichael.schiller@ehu.eus;

The supplementary information file contains:

- Fig. S1: O 1*s* and N 1*s* core level regions measured at the (111), (223) and (553) facets of the curved Rh(111) sample under 0.05 mbar NO at 25, 100 and 200°C
- Fig. S2: corresponding N 1*s* and Rh 3*d* α-scans obtained at the temperature onset of the NO dissociation (100°C for 0.05 mbar NO)
- Fig. S3: diffusion model of NO_{holl} towards the under-step reaction resulting into dissociation to O_{ads}
- Fig. S4: O 1*s* α-scan across the curved Rh(111) sample after surface oxidation at 200°C under 0.05 mbar NO
- Fig. S5: Real and reciprocal surfaces of Rh(111), Rh(223) and Rh(553)
- Fig. S6: Observed Bragg spots of Rh(111), Rh(223) and Rh(553)
- Fig. S7-11: Additional SXRD images obtained for Rh(111), Rh(553) and Rh(223)
- Fig. S12: Assessment of beam damage during AP-XPS experiments
- Video S1: Illustration of the α-scan approach combining curved crystals and AP-XPS

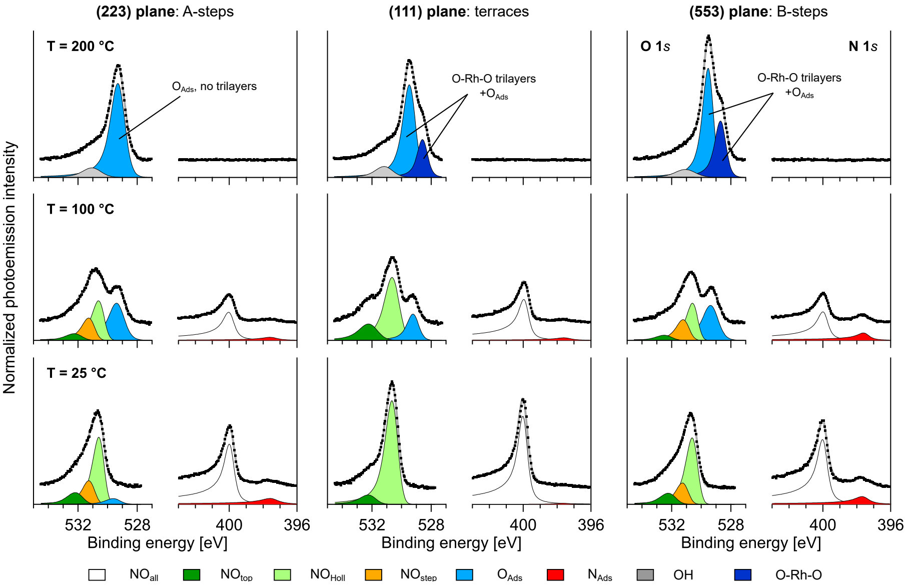

Fig. S1 O 1*s* and N 1*s* core levels for the (223), (111) and (553) surfaces in the c-Rh(111) crystal. Spectra were acquired during a heating ramp in 0.05 mbar NO (0.5 mbar mixture of 10% NO in He, flow of 1.5 ml/min, photon energy of 680 eV). The sample temperature was step-wise increased, and the photoemission spectra were acquired afterwards at the aforementioned facets. Annealing time per temperature point was roughly 90 min. O 1*s* spectra corresponds to data shown in Figures 1**a** and 2 of the main text. Due to final-state effects, individual species cannot be distinguished in the N 1*s* region, and only a single feature is observed (NO_{all}, ≈400 eV [1]). Moreover, atomic nitrogen (N_{ads}) is also detected at 397.3-397.8 eV [2]. While both N_{ads} and O_{ads} come from NO dissociation, the amount of N_{ads} is considerably lower. Similar to previous experiments [3], this is attributed to its removal via N+N or N+NO reactions. We observed a residual amount of carbon (amorphous "C", CO) contamination in the C 1*s* region (< 5% of the total NO signal, not shown). This contamination was maximum at 25°C, and progressively vanished from the surface upon heating in NO atmosphere.

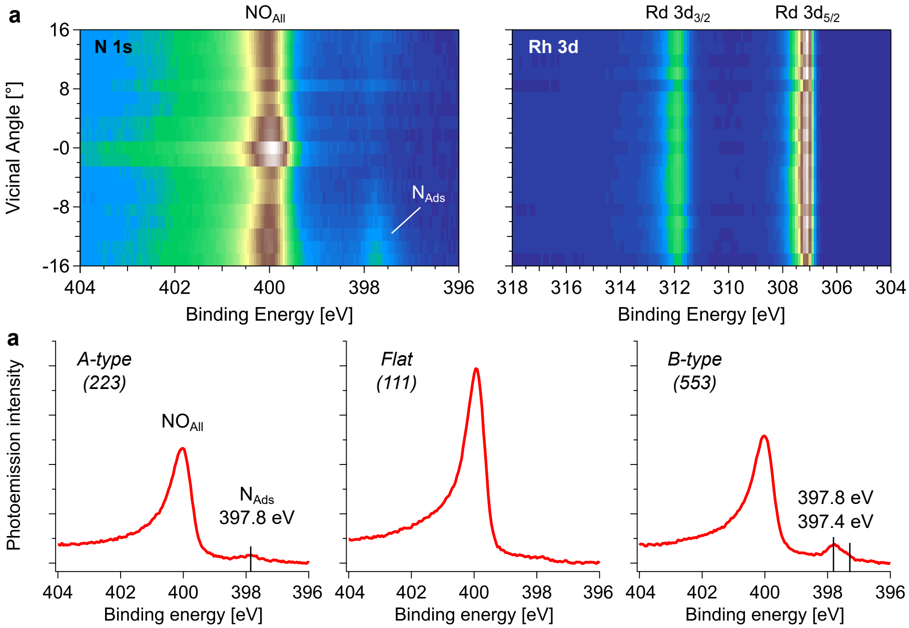

Fig. S2 **a** N 1s and Rh 3d α-scans acquired at 25°C after annealing the sample to 100°C in 0.05 mbar NO (0.5 mbar mixture of 10% NO in He) for 15 min and subsequent cooling to room temperature. Other components than Rh bulk atoms are difficult to distinguish in the Rh 3d region due to the relatively high photon energy of 680 eV. Data is complementary to the O 1s α-scan shown in Fig. 1**b**. The overall behavior of NO adsorbates is very similar for both the N 1s and O 1s α-scans even though their kinetic energy is ≈ 200 eV different, hence we discard photoelectron diffraction effects to occur across the curved surface. **b** Single N 1s spectra for the (223), (111) and (553) surfaces across the α-scan. A single contribution is observed for all NO species in the N 1s region (NO_{All}, ≈ 400 eV [1]). The peak arising from atomic nitrogen (N_{ads}) is detected at ≈ 397.8 eV for Rh(223), matching well with atomic nitrogen at steps. Both terrace and step components are detected for Rh(553) [2]. A very small emission is observed for Rh(111), which we attribute to defects at this surface.

Diffusion of NO_{holl} towards the under-step region.

The coverage variation of NO_{holl} and O_{ads} with α shown in the bottom panel of Fig. 1c remarkably departs from the expected linear evolution of terrace- and step-like adsorbates, as is the case of NO_{top} and NO_{step} shown on top of the same figure [4]. This may indicate that the NO_{holl} dissociation is not an on-site phenomenon, but it is rather governed by the ability of molecules to diffuse to active sites on the surface during annealing. For a diffusion length λ_{diff} that is significantly shorter than the average distance between active sites d, no dissociation is possible. On the contrary, for a much larger λ_{diff} the dissociation process is expected to affect all molecules. The probability *P(x)* for a molecule to diffuse to a distance x can be modeled into a first approach with the one-dimensional random walk function (see for example Ref. 5):

$$P(x) = \frac{1}{\lambda\sqrt{2\pi}} e^{-\frac{x^2}{2\lambda_{\mathrm{diff}}^2}} \tag{1}$$

where λ_{diff} would be a characteristic mean diffusion length for NO_{holl} molecules under the present NO dosing conditions (pressure, temperature, and dosing time). Fig. S3a represents *P(x)* for NO_{holl} molecules that diffuse from the terrace towards the next lower step at a distance d, where they dissociate. The relative probability for NO_{holl} molecules to reach the step is marked by the green shaded area. Therefore, the $O_{ads}(\alpha)$ coverage across the curved surface is given by:

$$\begin{aligned} O_{ads}(\alpha) &= \sigma_{dis} NO^0_{holl} \int_d^{\infty} P(x)dx \\ &= \sigma_{dis} NO^0_{holl} \left[ERFC\left(\frac{d(\alpha)}{\sqrt{2}\lambda_{\mathrm{diff}}}\right)\right] \end{aligned} \tag{2}$$

where ERFC stands for the complementary error function, $d(\alpha) = h/\sin(\alpha)$, σ_{dis} denotes the probability of the NO_{holl} molecule to dissociate when it reaches the step, and NO^0_{holl} represents a constant NO_{holl} saturation coverage across the curved surface at 100°C, right before the dissociation process begins. In Fig. S3b the read line fits the $O_{ads}(\alpha)$ data (red circles) with equation 2. The shaded area in each case contains most data points and their error bars, allowing us to estimate the range of variation of the fitting parameters. The fit renders $\lambda^A_{\mathrm{diff}} = 65\pm15$ Å and $\sigma^A_{dis} = 0.30\pm0.03$ for the steps of type A and $\lambda^B_{\mathrm{diff}} = 100\pm30$ Å and $\sigma^B_{dis} = 0.23\pm0.04$ for the vicinal surfaces of type B. This reflects that A-steps are more active than B-steps towards NO dissociation at its temperature onset, which is expected since the amount of oxygen at A-steps is slightly larger than at the B-side of the curved sample.

In Fig. S3b we also observe a qualitative correlation between the exponential $O_{ads}(\alpha)$ increase and the $NO_{\text{holl}}(\alpha)$ decrease away from the sample center (α=0), although we note that the $NO_{\text{holl}}(\alpha)$ intensity drop from the (111) center to the stepped edges is larger, compared to the intensity increase of $O_{ads}(\alpha)$. This is likely due to an additional NO_{holl} desorption process, which in fact coexists with dissociation at this temperature range [6–8]. Interestingly, to explain the $NO_{\text{holl}}(\alpha)$ variation in a quantitative way a similar α-dependent exponential increase in the NO_{holl} desorption rate must be considered. This suggests that the extra NO_{holl} desorption occurs near the steps and during the dissociation process, e.g, due to the increasing presence of O_{ads} covered areas that block the NO_{holl} adsorption nearby. In any case, we may still assume the same random-walk approach with the same λ_{diff} diffusion length to express the number of NO_{holl} molecules that remain on the surface after the 15 min/100°C flash as:

$$NO_{holl}(\alpha) = NO^0_{holl}\left[1 - (\sigma_{dis} + \sigma_{des})ERFC\left(\frac{d(\alpha)}{\sqrt{2}\lambda_{\text{diff}}}\right)\right] \tag{3}$$

where σ_{des} represents an effective NO_{holl} desorption probability induced by the dissociation process near the step. Fitting the $NO_{\text{holl}}(\alpha)$ data points with Eq. 3 (solid black lines) renders $\sigma^A_{des} = 0.47\pm0.03$ and $\sigma^B_{des} = 0.38\pm0.03$ for A and B steps, respectively. Thus, steps cause a similar but slightly lower dissociation/desorption ratio during the present AP-XPS experiment, as compared to values obtained during the annealing of NO-saturated layers in UHV [6–8].

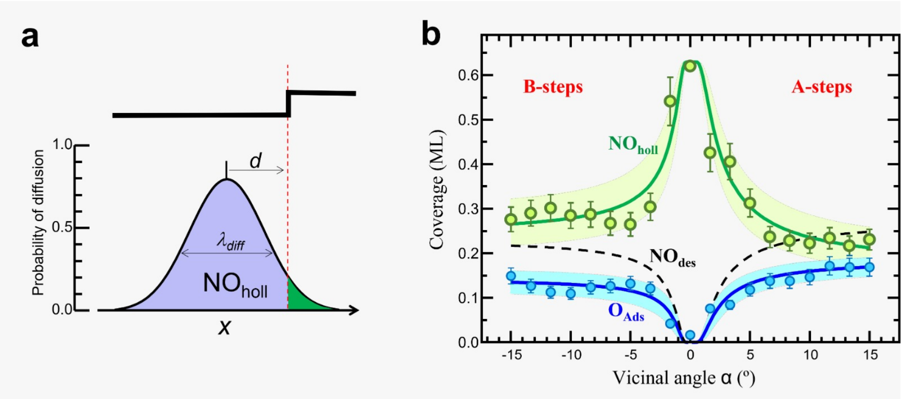

Fig. S3 **a** Probability (shaded green area) for a NO_{holl} molecule to reach a step that is located at a distance d, determined by the random walk function $P(x)$ and the diffusion length λ_{diff}. **b** Fitting curves for the $NO_{\text{holl}}(\alpha)$ and $O_{\text{ads}}(\alpha)$ data of Fig. 1c using Eq. 2 and 3 (black and red, respectively). The fit requires the variable $NO_{\text{des}}(\alpha)$ desorption represented with the dashed blue line, i.e., both dissociation $O_{\text{ads}}(\alpha)$ and desorption $NO_{\text{des}}(\alpha)$ probabilities explain the remaining $NO_{\text{holl}}(\alpha)$ coverage. Shaded areas around fitting lines contain most data points and their error bars. They indicate the range within which fitting parameters have been varied, allowing us to estimate their degree of accuracy

.

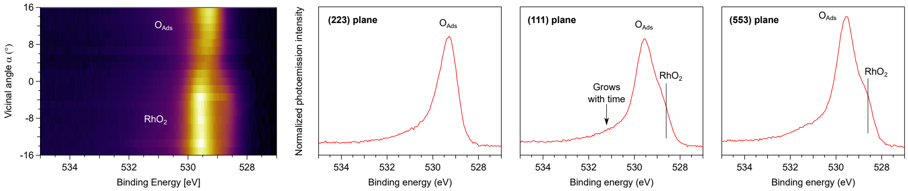

Fig. S4 O 1s α-scan acquired at 200°C in 0.05 mbar NO (0.5 mbar mixture of 10% NO in He) at 680 eV photon energy. As mentioned in the text, it is clear that the oxygen in the A-side of the crystal remains chemisorbed, while that of the (111) terraces and the B-type forms the surface oxide. We observed the growth of the small shoulder at 531 eV with time at the (111) surface (not shown). Further oxidation of Rh towards Rh_2O_3 is not likely under these conditions, although it could be induced by the intense X-ray beam and would explain the peak growth with time [9]. The binding energy also matches well with adsorbed OH arising from water contamination in the chamber, yet one would expect water and OH desorption at lower temperatures.

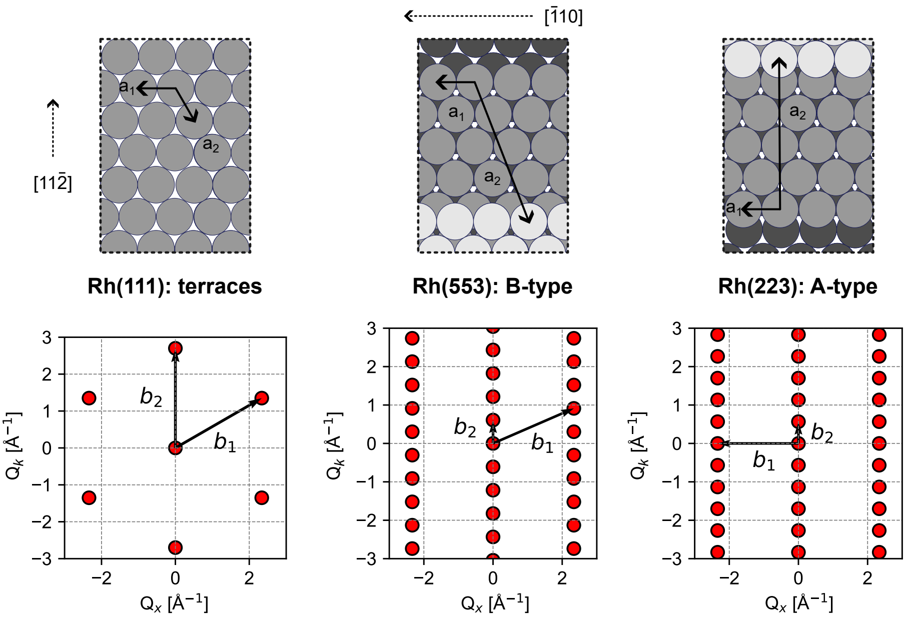

Fig. S5 Real (top row) and reciprocal (bottom row) surface lattices for Rh(111), Rh(553) and Rh(223), showing both real ($\mathbf{a_1}$ and $\mathbf{a_2}$) and reciprocal ($\mathbf{b_1}$ and $\mathbf{b_2}$, namely h and k) in-plane vectors. On one hand, the small $\mathbf{a_1}$ is defined for the three surfaces along the $[\bar{1}10]$ direction, which coincides with the step edge for vicinal surfaces. While $\mathbf{a_1}$ and $\mathbf{a_2}$ are symmetrically equivalent for Rh(111), $\mathbf{a_2}$ accounts for the terrace width in stepped surfaces, thereby $\mathbf{a_1}$ and $\mathbf{a_2}$ are not symmetrically equivalent in stepped surfaces and the reciprocal in-plane $\mathbf{b_1}$ and $\mathbf{b_2}$ vectors provide different information. See Methods for more details about the $\mathbf{a_i}$ and $\mathbf{b_i}$ vectors of the three surfaces.

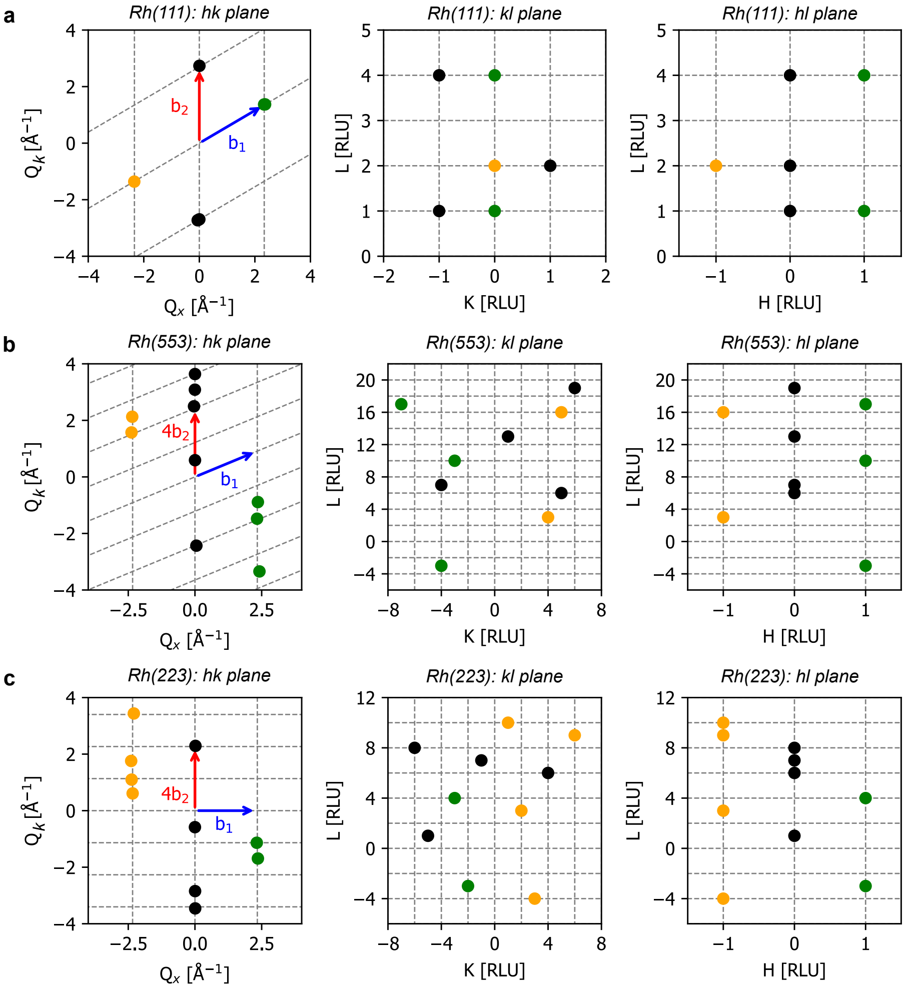

Fig. S6 Observed Bragg spots in SXRD for the **a** Rh(111), **a** Rh(553) and **c** Rh(223) samples. Scattering Q-coordinates were aligned so that Q_k and Q_l match the direction of k ($\mathbf{b_2}$) and l ($\mathbf{b_3}$), respectively. The vector Q_x is defined perpendicular to Q_k, and matches the direction of h if the in-plane vectors are orthogonal. Otherwise, h has both Q_x and Q_k components and spans along Q_r. The corresponding Q_xQ_k planes for the surfaces are shown in the first column of the Figure. Q_r, Q_k and Q_l scattering vectors were normalized by h, k and l to transform Q-coordinates to relative lattice units (RLU). In the second and third column we show the kl and hl planes after the conversion. Bragg spots are colored depending on their H value, following green for H=1, yellow for H=-1, and black for H=0 (K-direction). Reciprocal space alignment is thoroughly described in Methods.

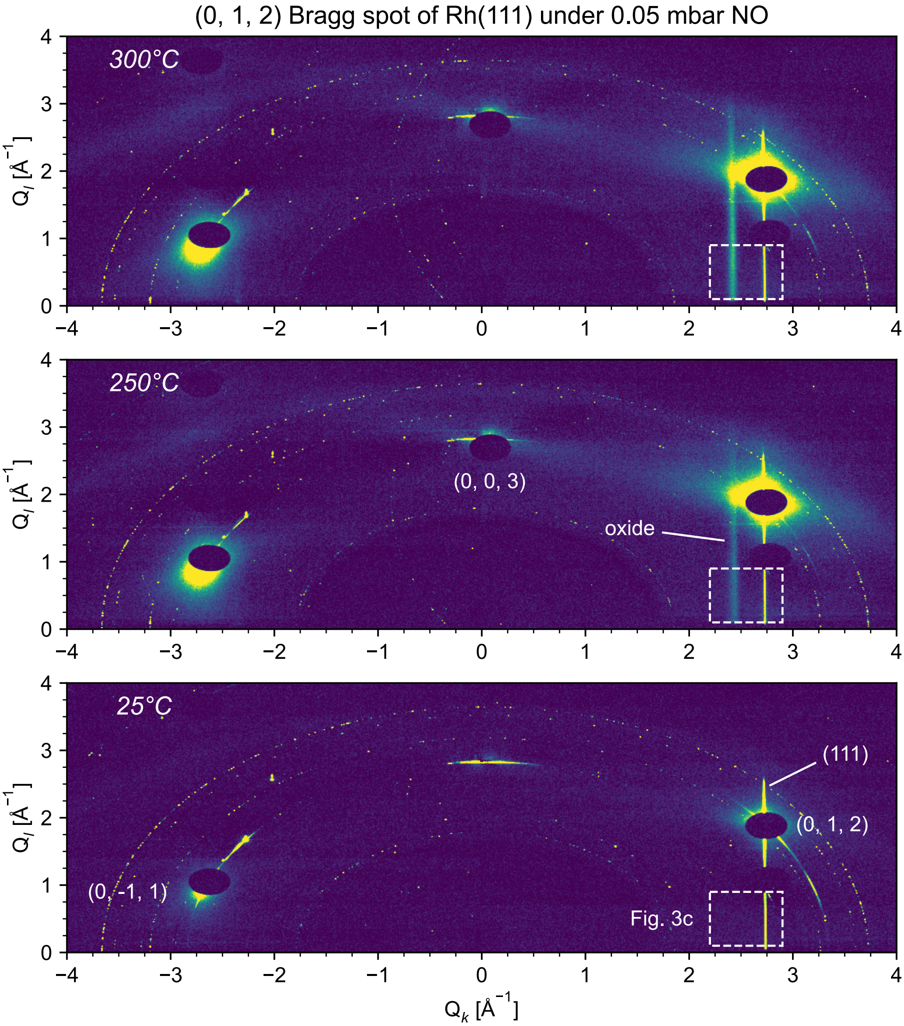

Fig. S7 SXRD images along the k-direction of Rh(111) obtained using a flat single crystal. The entire image from which the data of Fig. 3c is extracted is shown. Images were acquired under 0.05 mbar NO at 68 keV, before (25°C), at the onset of (250°C), and after (300°C) surface oxide formation. The dashed rectangle marks the area of Fig. 3c, showing how the intensity arises from the (0, 1, 2) Bragg spot. The (-1, 0, 1) and (0, 0, 3) Bragg spots are also seen. All Bragg spots observed for the Rh(111) sample are shown in Fig. S6**a**. Powder diffraction rings are also detected in the image. On the one hand, the rings that do not intersect the Bragg spots likely stem from the Be areas of the chamber. On the other hand, small powder rings intersecting the Bragg spots probably arise from disordered Rh areas, although the large intensity of the (111) rod indicates that Rh(111) is well-ordered under these conditions.

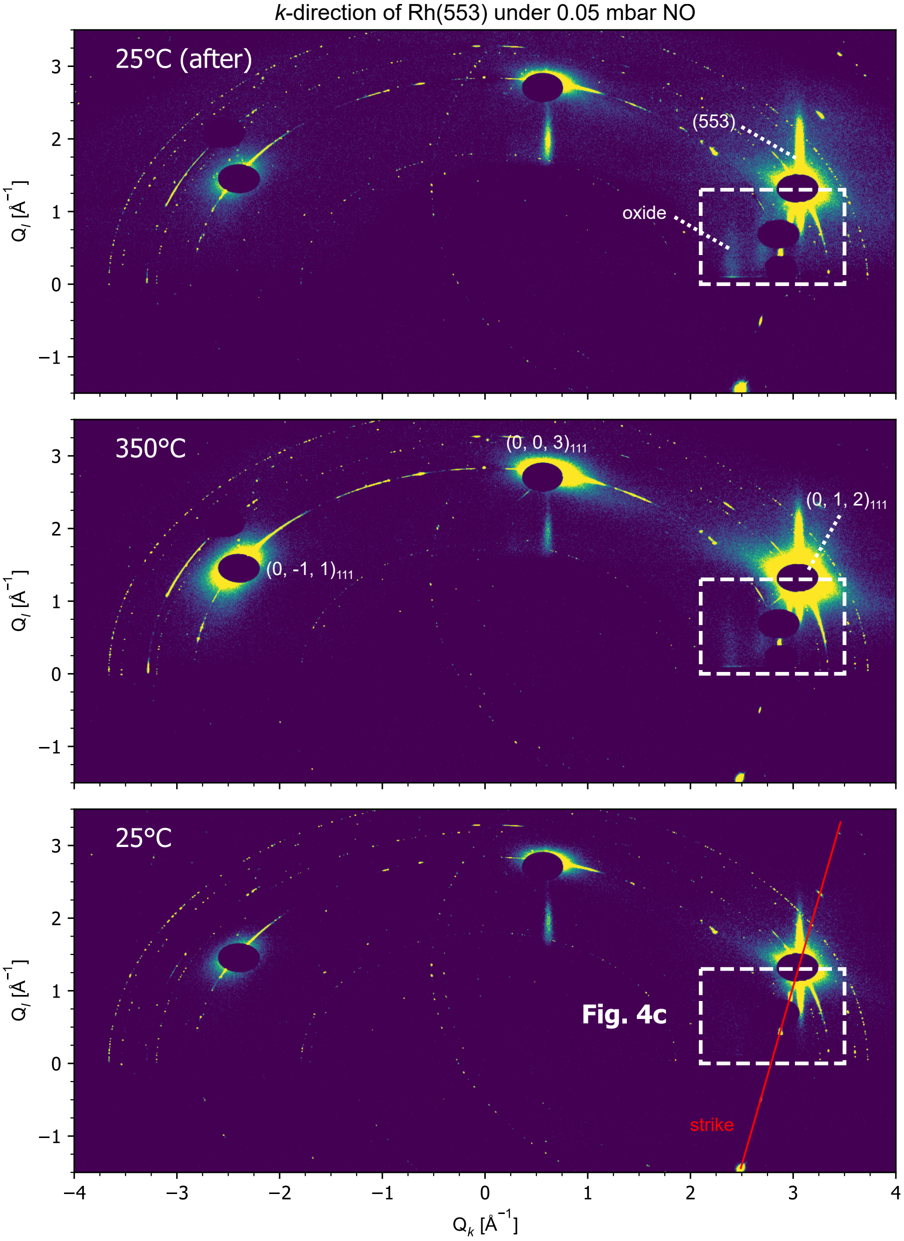

Fig. S8 SXRD images along the k-direction of Rh(553) obtained using a flat single crystal, showing the entire image from which the data of Fig. 4c is extracted. SXRD images were acquired at 68 keV after oxidizing the crystals under 0.05 mbar NO at 25°C after dosing the gases, at 350°C, and again at 25°C after annealing the sample. The dashed rectangle marks the area shown in Fig. 4c, where the intensity arises from the $(0, 1, 2)_{111}$ Bragg spot. The $(0, -1, 1)_{111}$ and $(0, 0, 3)_{111}$ Bragg spots belonging to the k-direction are observed as well. All Bragg spots of Rh(553) are shown in Fig. S6b, and powder rings are described in Fig. S7 above. As mentioned in the main text, diffraction forming a strike is likely arising from multiple scattering effects which are outside of the focus of this publication.

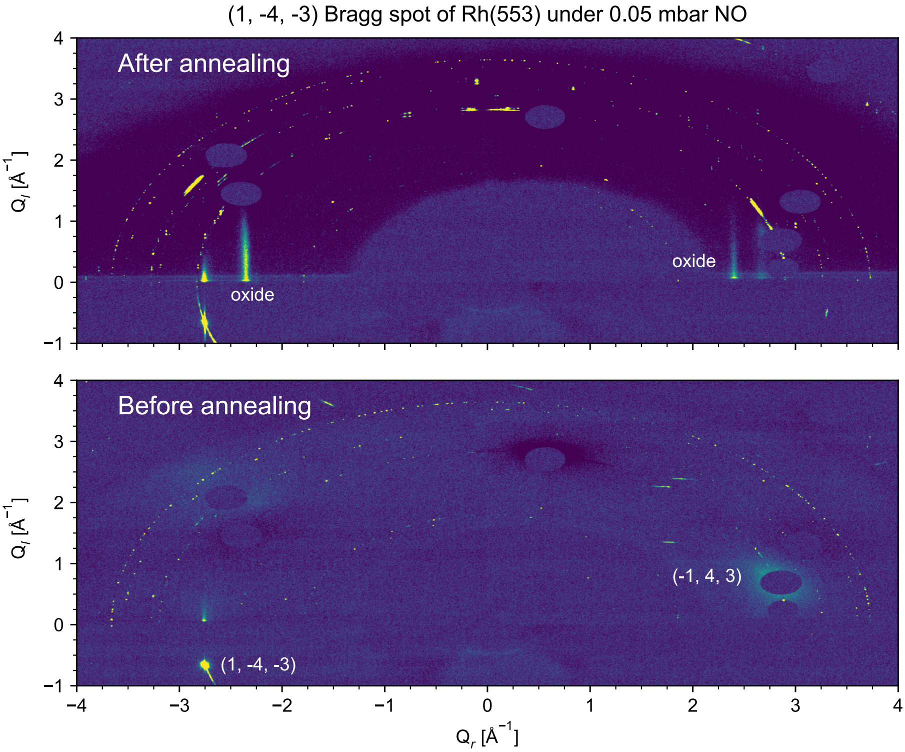

Fig. S9 SXRD images arising from the (1, -4, -3) Bragg spot of Rh(553) obtained using a flat single crystal. Images were acquired under 0.05 mbar NO at 68 keV at 25°C before (no oxide) and after annealing (oxidized surface) to 350°C. In this case, intensity arises from the (1, -4, -3) Bragg spot, which is rotated roughly 120° from the k-direction of Rh(553) shown in Fig. S8. All Bragg spots of Rh(553) are shown in Fig. S6b, and powder rings are described in Fig. S7 above. The data before oxidation show a single rod corresponding to a CTR extending from the (1, -4. -3) Bragg reflection of Rh(553). After oxidation, extra vertical rods are found at about $Q_r \approx 2.4$ and -2.4 Å^{-1}, in agreement with the growth of the surface oxide described for Rh(111) [10].

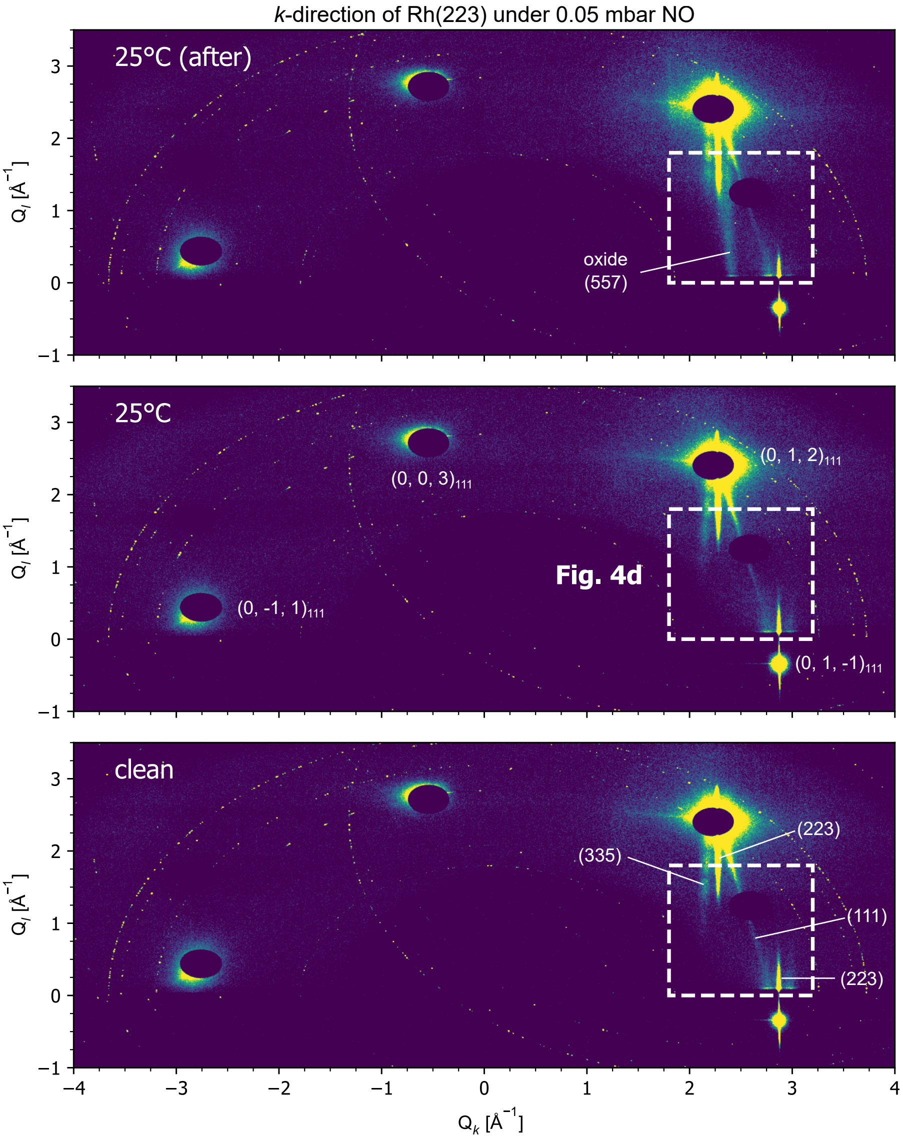

Fig. S10 SXRD images along the k-direction of Rh(223) obtained using a flat single crystal, showing the entire image from which the data of Fig. 4d is extracted. Images were acquired after cleaning the sample in UHV and under 0.05 mbar NO at 68 keV at 25°C before (no oxide) and after annealing (oxidized surface) to 350°C. The same leaning rods are observed after cleaning and after exposing the gases without annealing, therefore the gas exposure does not cause the faceting of Rh(223). The dashed rectangle marks the area shown in Fig. 4d, where the intensity arises from the $(0, 1, 2)_{111}$ and $(0, 1, \text{-}1)_{111}$ Bragg spots. The $(0, \text{-}1, 1)_{111}$ and $(0, 0, 3)_{111}$ Bragg spots belonging to the k-direction are observed as well. All Bragg spots observed for the Rh(223) sample are shown in Fig. S6c, and powder rings are described in Fig. S7 above.

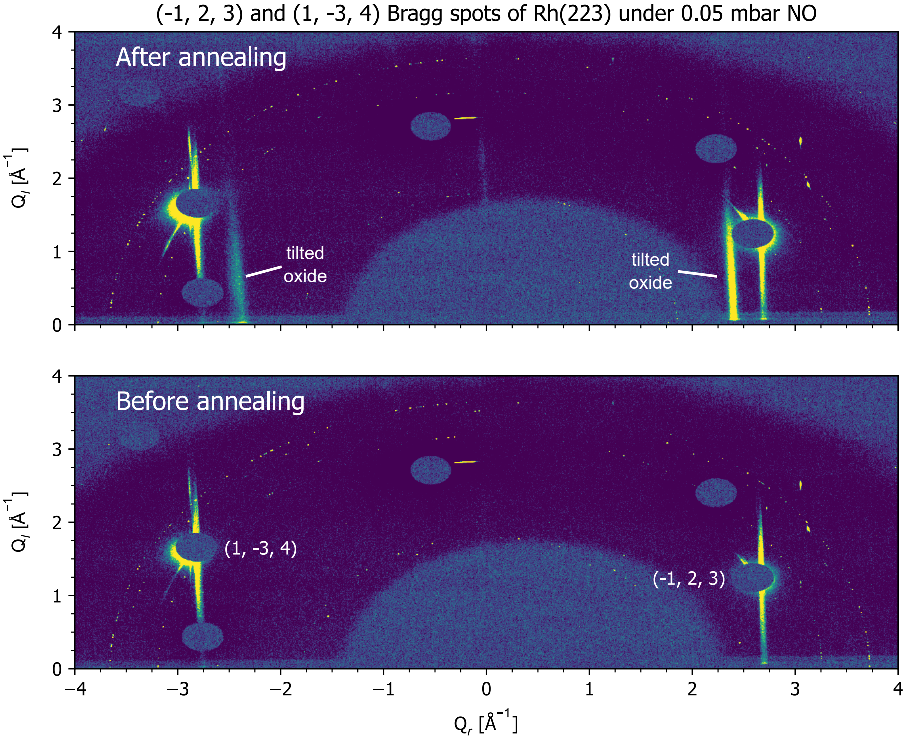

Fig. S11 SXRD images arising from the (-1, 2, 3) and (1, -3, 4) Bragg spotd of Rh(223) obtained obtained using a flat single crystal. Images were acquired under 0.05 mbar NO at 68 keV at 25°C, before (no oxide) and after annealing (oxidized surface) to 350°C. In this case, intensity arises from the (1, -3, 4) and (-1, 2, 3) Bragg spots rotated roughly 120° from the *k*-direction of Rh(223) shown in Fig. S10. Before surface oxidation, two leaning CTRs are observed at $Q_r \approx 2.7$ and -2.7 Å^{-1}. After oxidation, additional surface oxide rods appear at $Q_r \approx 2.4$ and -2.4 Å^{-1}. All Bragg spots observed for the Rh(223) sample are shown in Fig. S6c, and powder rings are described in Fig. S7 above.

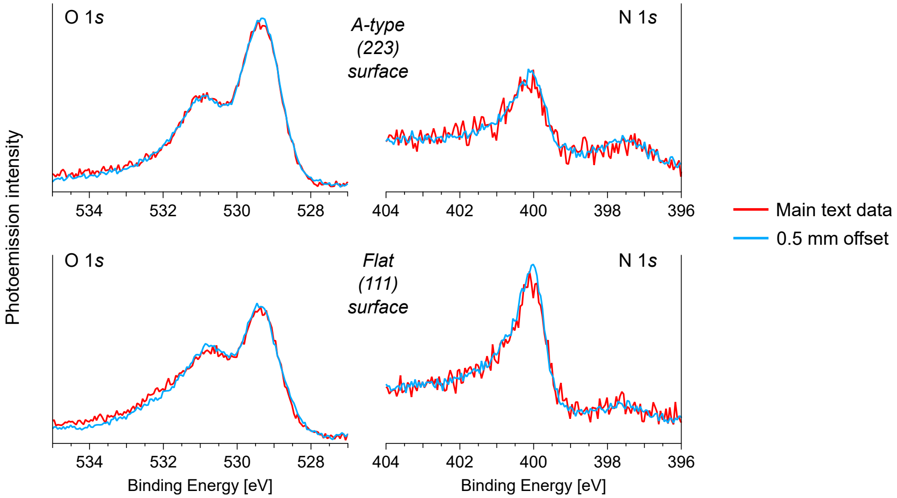

Fig. S12 Spectra at different horizontal positions at (223) and (111) planes. O 1*s* and N 1*s* core levels for the (223) and (111) surfaces at 100°C. Consecutive spectra were acquired at a different horizontal position but at the same vicinal angle α to check for beam damage. As easily observed, the spectra in the new point (red line, acquisition time < 3 min) is almost identical as the ones shown in Fig. 1a and 2b (blue lines, acquisition time ≈ 10 min and hence better statistics). Therefore, we do not observe a sizable beam damage during this interval. The flow was 1.5 ml/min, the total pressure was 0.5 mbar (10% NO diluted on He), the photon energy was 680 eV.

References

[1] F.G. Requejo, E.L. Hebenstreit, D.F. Ogletree, M. Salmeron, An in situ XPS study of site competition between CO and NO on Rh(111) in equilibrium with the gas phase. Journal of Catalysis **226**(1), 83–87 (2004). https://doi.org/10.1016/j.jcat.2004.05.010

[2] F. Esch, A. Baraldi, C. Comelli, S. Lizzit, M. Kiskinova, P.D. Cobden, B.E. Nieuwenhuys, Atomic nitrogen on steps: A fast x-ray photoelectron spectroscopy study of the NO uptake on Rh(533), Rh(311), and Rh(111). Journal of Chemical Physics **110**(8), 4013–4019 (1999). https://doi.org/10.1063/1.478282

[3] L. Rämisch, R. Temperton, S.M. Gericke, S. Pfaff, A. Shavorskiy, E. Lundgren, J. Zetterberg, F. García-Martínez, Multi modal time-resolved infrared and X-ray spectroscopic operando studies of the CO oxidation and NO reduction reactions on Rh(111). Applied Surface Science **687**, 161,989 (2025). https://doi.org/https://doi.org/10.1016/j.apsusc.2024.161989

[4] F. Garcia-Martinez, F. Schiller, S. Blomberg, M. Shipilin, L.R. Merte, J. Gustafson, E. Lundgren, J.E. Ortega, CO chemisorption on vicinal Rh(111) surfaces studied with a curved crystal. The Journal of Physical Chemistry C **124**(17), 9305–9313 (2020). https://doi.org/10.1021/acs.jpcc.0c00039. URL https://doi.org/10.1021/acs.jpcc.0c00039

[5] J.B. Keller, Diffusion at finite speed and random walks. Proceedings of the National Academy of Sciences **101**(5), 1120–1122 (2004). https://doi.org/10.1073/pnas.0307052101

[6] D. Castner, G. Somorjai, LEED and thermal desorption studies of small molecules (H_2, O_2, CO, CO_2, NO, C_2H_4, C_2H_2 and C) chemisorbed on the stepped Rhodium (755) and (331) surfaces. Surface Science **83**(1), 60–82 (1979). https://doi.org/https://doi.org/10.1016/0039-6028(79)90480-1

[7] L.A. DeLouise, N. Winograd, Adsorption and desorption of NO from Rh111 and Rh331 surfaces. Surface Science **159**(1), 199–213 (1985). https://doi.org/https://doi.org/10.1016/0039-6028(85)90112-8

[8] The dissociation kinetics of NO on Rh(111) as studied by temperature programmed static secondary ion mass spectrometry and desorption. The Journal of Chemical Physics **101**(11), 10,052–10,063 (1994). https://doi.org/10.1063/1.467994

[9] F. Schiller, K. Ali, A.A. Makarova, S.V. Auras, F. García-Martínez, A. Mohammed Idris Bakhit, R. Castrillo Bodero, I.J. Villar-García, J.E. Ortega, V. Pérez-Dieste, Near-Ambient Pressure Oxidation of Silver in

the Presence of Steps: Electrophilic Oxygen and Sulfur Impurities. ACS Catalysis **14**(17) (2024). https://doi.org/10.1021/acscatal.4c02985

[10] J. Gustafson, A. Mikkelsen, M. Borg, E. Lundgren, L. Köhler, G. Kresse, M. Schmid, P. Varga, J. Yuhara, X. Torrelles, C. Quirós, J.N. Andersen, Self-limited growth of a thin oxide layer on Rh(111). Physical Review Letters **92**(12), 10–13 (2004). https://doi.org/10.1103/PhysRevLett.92.126102
